# Acupuncture combined with moxibustion mitigates spinal cord injury-induced motor dysfunction in mice by NLRP3-IL-18 signaling pathway inhibition

**DOI:** 10.1186/s13018-023-03902-6

**Published:** 2023-06-09

**Authors:** Ji-Hui Zheng, Na Yuan, Peng Zhang, De-Feng Liu, Wei Lin, Jun Miao

**Affiliations:** 1 Department of OrthopaedicsThe Graduate School, Tianjin Medical University, Tianjin, China; 2 Hebei Key Laboratory of Integrated Traditional and Western Medicine in Osteoarthrosis Research (Preparing), Cangzhou, China; 3 Department of Orthopaedics, Hebei Province Cangzhou Hospital of Integrated Traditional and Western Medicine, Cangzhou, China; 4 Department of OrthopaedicsTianjin Hospital, Tianjin Medical University, Tianjin, China

**Keywords:** Spinal cord injury, Acupuncture, Moxibustion, Astrocyte, Interleukin-18, NLRP3

## Abstract

**Background:**

Spinal cord injury (SCI), which reportedly induces severe motor dysfunction, imposes a significant social and financial burden on affected individuals, families, communities, and nations. Acupuncture combined with moxibustion (AM) therapy has been widely used for motor dysfunction treatment, but the underlying mechanisms remain unknown. In this work, we aimed to determine whether AM therapy could alleviate motor impairment post-SCI and, if so, the potential mechanism.

**Methods:**

A SCI model was established in mice through impact methods. AM treatment was performed in SCI model mice at Dazhui (GV14) and Jiaji points (T7-T12), Mingmen (GV4), Zusanli (ST36), and Ciliao (BL32) on both sides for 30 min once per day for 28 days. The Basso–Beattie–Bresnahan score was used to assess motor function in mice. A series of experiments including astrocytes activation detected by immunofluorescence, the roles of NOD-like receptor pyrin domain-containing-3 (NLRP3)–IL-18 signaling pathway with the application of astrocyte-specific NLRP3 knockout mice, and western blot were performed to explore the specific mechanism of AM treatment in SCI.

**Results:**

Our data indicated that mice with SCI exposure exhibited motor dysfunction, a significant decrease of neuronal cells, a remarkable activation of astrocytes and microglia, an increase of IL-6, TNF-α, IL-18 expression, and an elevation of IL-18 colocalized with astrocytes, while astrocytes-specific NLRP3 knockout heavily reversed these changes. Besides, AM treatment simulated the neuroprotective effects of astrocyte-specific NLRP3 knockout, whereas an activator of NLRP3 nigericin partially reversed the AM neuroprotective effects.

**Conclusion:**

AM treatment mitigates SCI-induced motor dysfunction in mice; this protective mechanism may be related to the NLRP3–IL18 signaling pathway inhibition in astrocytes.

**Supplementary Information:**

The online version contains supplementary material available at 10.1186/s13018-023-03902-6.

## Introduction

Spinal cord injury (SCI), one of the most devastating central nervous system (CNS) injuries, often results in irreversible motor and neurosensory dysfunction, greatly impairing a patient's quality of life [1–4]. Motor dysfunction has been reported to be one of the most frequent complications of neurological dysfunction in patients after SCI [5]. Little is known about effective interventions for patients with motor dysfunction after spinal cord injury, highlighting the need for research into the underlying mechanisms of this complication and new effective therapies development to alleviate symptoms in this patient population.

The SCI pathogenesis is complex and can be divided into two phases. After a brief primary injury, persistent secondary injury, dominated by neuroinflammation, leads to disruption of the blood-spinal cord barrier, neuronal damage, and demyelination, resulting in axonal destruction [6, 7]. Previous studies showed that SCI-induced neuroinflammation, which leads to progressive neurodegeneration and neuronal loss, is closely associated with motor and neurosensory dysfunction [8, 9]. Recently, several studies suggested that NLRP3-mediated pyroptosis plays an irreplaceable role in the inflammatory response after SCI [9, 10]. Targeting NLRP3 inflammasome components has been demonstrated significantly to mitigate SCI-induced motor dysfunction through inflammatory response inhibition [10]. The NLRP3 inflammasome is reported to activate and then recruit caspase-1 and apoptosis-associated speckle-like protein (ASC), leading to the release of pro-inflammatory cytokines IL-18 [11].

Interestingly, it was demonstrated that IL-18 had been shown to impact immune response initiation and adaptability, leading to aggravating inflammatory response post-SCI [10]. Besides, persistent inflammatory stimuli boost the IL-18–IL-18R signal in reactive astrocytes, subsequently producing several inflammatory proteins, such as IL-6 and IL-1β [12, 13]. Tohda C et al. also reported that the reactive astrocytes activation that contributes to glial scar formation participates in the SCI process [14]. However, glial scarring as a barrier to neural regeneration inhibits axonal regeneration and ultimately hinders the recovery of nervous system function [15, 16]. A previous study has shown that the inhibition of astrocytic activation promotes motor recovery after SCI [17]. Until now, the role of NLRP3–IL-18 signals in astrocytes under the SCI condition remains ambiguous.

Previous research has shown that moxibustion or electroacupuncture can modulate the inflammatory response, promote nerve cell proliferation, enhance neuronal growth and nerve regeneration, and accelerate motor function recovery in SCI patients [18–20]. Acupuncture involves inserting a needle into the skin to stimulate the acupoints. In contrast, moxibustion uses infrared light and heat created by burning folium artemisiae argyi to activate acupoints. Recently, acupuncture combined with moxibustion (AM) has been used as an alternative treatment for various diseases [21, 22]. According to the idea of Zang-fu organs and meridians in traditional Chinese medicine, damage to the "Governor Vessel" might explain the pathological alteration in SCI [23]. The "Governor Vessel" is a back circulation meridian whose location aligns with the spinal cord. Therefore, to encourage the restoration of meridian function, we selected the acupoints situated in the "Governor Vessel," such as Dazhui (GV14), Jiaji points (T7-T12), and "Mingmen" (GV4). Zusanli (ST36) and Ciliao (BL32) have the potential to improve blood circulation and muscular function rehabilitation [18]. Nigericin is a Streptomyces hygroscopicus-derived antibiotic that serves as a K^+^/H^+^ ionophore [24]. It promotes K^+^/H^+^ exchange across mitochondrial membranes and can activate NLRP3, which leads to the release of IL-18 in a NALP3-dependent manner [25, 26]. According to our knowledge, few studies have examined the link between the NLRP3–IL18 signaling pathway and AM in SCI patients.

In this study, we aimed to determine if modifying the NLRP3–L18 signaling pathway might be a potential treatment strategy for improving motor dysfunction post-SCI. We sought to determine whether AM treatment ameliorated motor dysfunction post-SCI by inhibiting the NLRP3–IL18 signaling pathway.

## Materials and methods

### Animals

The Institutional Animal Care and Use Committee of the Hebei Province Cangzhou Hospital of Integrated Traditional and Western Medicine approved each experimental procedure. The current study's protocols and details were completed following the Animal Research: Reporting of In Vivo Experiments (ARRIVE) criteria. Moreover, the information on key resources, including antibodies, reagents, and software, is provided in Additional file 1: Table S1. Male C57BL/6 mice and astrocytes-specific NLRP3 knockout mice weighing 25–28 g, were used to establish SCI model. Prior to surgical treatment, mice were kept in standard cages with regulated temperatures (24 ± 1 °C) separately, 12-h light/dark cycles, with water and food ad libitum. In the first stage, wild-type mice were randomly assigned to two groups: (1) Sham (*n* = 12), (2) SCI (*n* = 12); in the second stage, wild type mice and NLRP3-KO mice were selected and divided into two groups: (1) wild-type (WT) (*n* = 12), (2) NLRP3-KO (*n* = 15). in the third stage, wild-type mice were randomly assigned to four groups: (1) SCI + AM + Vehicle (*n* = 12), (2) SCI + AM + Nigericin (*n* = 12), (3) SCI + Control (Con) + Vehicle (*n* = 12), (4) SCI + Control (Con) + Nigericin (*n* = 12).

### SCI model

**Fig. 1:**
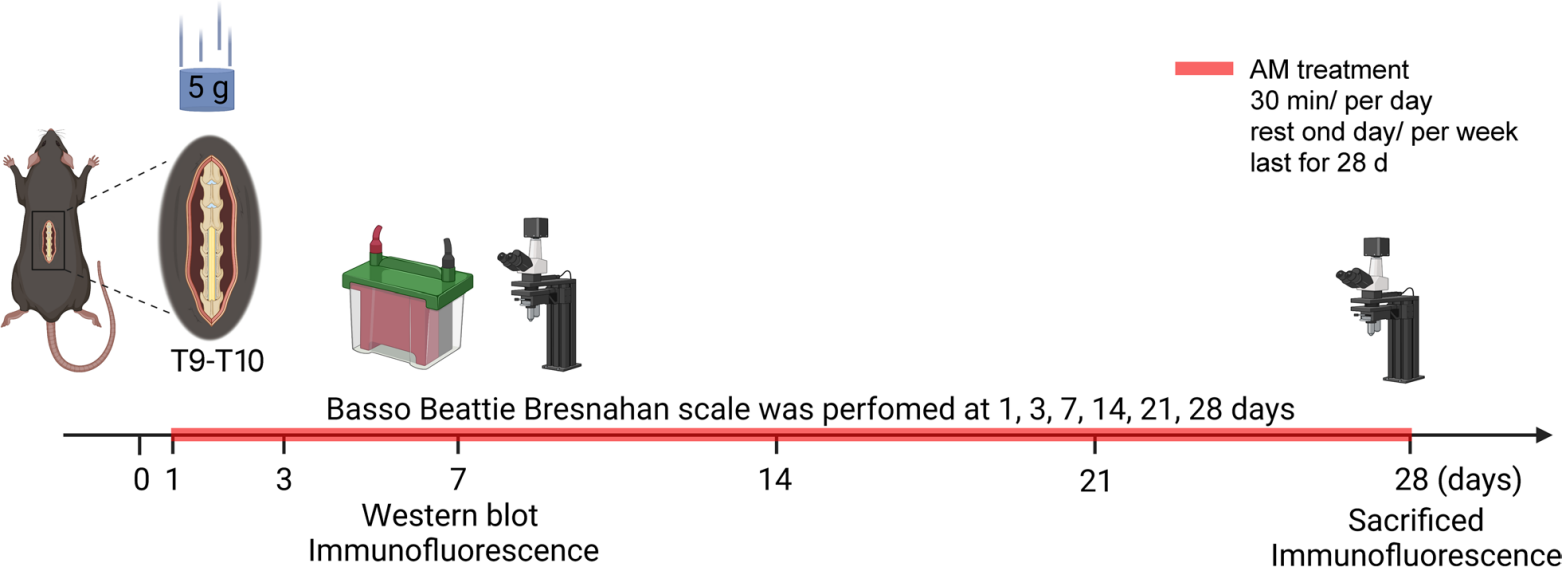
Flow diagram of the study procedure. (Created with Biorender.com). AM treatment (Acupuncture combined with moxibustion treatment)

Under sevoflurane anesthesia (induction 7–8%, maintenance 3–4%), mice related to temperature and electrocardiogram monitoring. The body temperature was maintained at 37 ± 1 °C by a heating blanket. To reveal the spinal cord at T9-T10, a laminectomy was conducted. A 5 g weight impactor was dropped from a height of 5 cm onto the exposed dorsal surface of the spinal cord for contusion (Fig. 1) [27]. The dwell time was 0.5 s, and the compression was 1.5 mm. After rinsing the wound with sterile saline and inserting an absorbable gelatin sponge, the muscle and skin were stitched in layers. Mice exhibited the following characteristics, demonstrating a successful model: subdural hemorrhage; the muscles of the lower limbs, head, and neck briefly contracted several times; moreover, vigorously wag the mice tail for a few seconds. This injury was moderate and resulted in incomplete paraplegia in mice. The mice in the sham-operated group were only performed with spines and nerve plates removed but without no spinal cord injury. To avoid postoperative infection, gentamicin (8 mg/kg) was administered intramuscularly daily for three days following SCI. Manual bladder pressure was applied twice daily to squeeze urine until autonomous bladder activity was restored (to prevent urinary retention).

### Drug administration

For intrathecal injection (IT), the animal was briefly anesthetized with sevoflurane. Nigericin (5 μg/5 μL) was given 1 h before the injury through intrathecal injection. The dissolving reagent for nigericin (5 μl) was progressively injected into the subarachnoid space of mice between the L5 and L6 vertebrae using a needle connected to a microsyringe [28, 29]. About 90% maize oil and 10% ethyl alcohol made up the reagent for nigericin's dissolving. An equivalent amount of dissolved reagent was injected intrathecally as a vehicle for the therapy.

### Acupuncture and moxibustion

Acupuncture combined with moxibustion (AM) therapy was applied to the SCI mice. Dazhui (GV14), Jiaji points (T7-T12), "Mingmen" (GV4), Zusanli (ST36), and Ciliao (BL32) of both sides were chosen [18, 30, 31]. Under sevoflurane anesthesia, those acupoints were punctured with stainless 0.18 mm-diameter needles inserted around 5 mm deep. Meanwhile, Dazhui (GV14), Mingmen (GV4), and Zusanli (ST36) were treated by moxibustion (Made by Hebei Province Cangzhou Hospital of Integrated Traditional and Western Medicine, Cangzhou, China). Once daily, each treatment lasted for 30 min. Mice in the treatment groups received therapy on the first day after SCI with one day of rest per week for 28 days (Fig. 1). The control treatment was performed by sevoflurane anesthesia only.

### Basso Beattie Bresnahan (BBB) scale

To measure hindlimb motor function, BBB was performed in mice at 1, 3, 7, 14, 21 and 28 days in an open field after SCI (Fig. 1) [32]. Two researchers who were blind to the group assignment conducted the measurement. Mice were permitted to acclimate to the testing environment. After the mice had adapted to the environment, experimenters observed and scored the locomotor function in 5 min. The scores range from 0 to 21: 0 point, the hindlimbs were paralyzed; 1–7 points, the hindlimb joints were limited in their range of motion; 8–13 points, the mice could walk to a certain extent beside the hindlimb joints can move; 14–20 points, the mice could perform fine movements with their paws; 21 point, the mice motor function was completely normal.

### Immunofluorescence

Sevoflurane 7–8% was utilized to anesthetize the mice before perfusing with normal saline and 10% neutral buffer formalin by the left cardiac apex. The tissues were fixed with 10% neutral buffer formalin for 48 h and dehydrated with graded alcohol after carefully removing the spinal cord tissue samples. The tissue was then embedded in paraffin and coronally cut into 4 μm slices after being incubated with xylene. After being hydrated, the sections underwent an antigen retrieval incubation with sodium citrate at 0.15 MP for 5 min. The sections were infiltrated for 20 min with 0.1% Triton, then sealed for 1 h with a blocking solution. The sections were incubated with a primary monoclonal rabbit antibody against NeuN (1:200), a primary polyclonal rabbit antibody against IL-18 (1: 200), NLRP3 (1: 100), a monoclonal mouse antibody against the glial fibrillary acidic protein (GFAP) (1:500) and a polyclonal goat antibody against Iba1 (1:200) at 4 °C overnight. The secondary antibodies included Cy3-labeled donkey anti-goat IgG (1:500), CyTM3-conjugated goat anti-mouse IgG (1:500), FITC-conjugated goat anti-Rabbit IgG (1:500). The nuclei were identified by 4′-6′-diamino-2-phenylindole (DAPI). The same conditions were used for all immunostaining processes to reduce sample heterogeneity. Images were taken using a fluorescence microscope with a motorized stage. The optical fractionator method offered by the Stereo Investigator software version 9.0 was used to count immunoreactive cells on a 1/10 series of 40 μm coronal slices. Four 3.7 mm^2^ 400 × fields of view were randomly selected under the microscope, and the mean values were calculated. ImageJ was used to examine the number of double-labeled cells and the fluorescence intensity in the designated region.

### Western Blot

Seven-day post-SCI, mice were anesthetized with 7–8% sevoflurane and perfused with cold saline by the left cardiac apex. The spinal cord tissues from each group were carefully isolated under a stereomicroscope and cut into pieces in the cell lysis buffer. After centrifuging at 12,000 × g for 5 min, the supernatant was recovered. After the bicinchoninic acid assay determined protein concentration, the sample (30 μg) was separated by 10% SDS-PAGE and then transferred onto a PVDF membrane. After sealing with Quickblock at 25 °C for 10 min, the PVDF membranes were incubated with polyclonal rabbit anti-IL-18, monoclonal mouse anti-IL-6, and polyclonal rabbit anti-TNF-α overnight at 4 °C. The next day, sections were incubated with goat anti-rabbit or goat anti-mouse secondary antibody at 25 °C for 1 h. Image Lab identified and analyzed the protein bands after enhanced chemiluminescence (ECL) incubation. α-Tubulin was used as the internal reference protein.

### Statistical analysis

According to our preliminary results, SAS 9.1 software was utilized to discover statistical differences using a 5% significance level test (*α* = 0.05) with a power of 80% (*β* = 0.2). All statistical calculations were conducted using GraphPad Prism 8.0.1 software. For testing the data's normality, the Shapiro–Wilk test was applied. Levene's test was used to examine whether the variance was homogeneous. Once data heteroscedasticity was found, it was fixed using a logarithmic data transformation. For comparing the groups, one-way or two-way analysis of variance (ANOVA) along with Tukey post hoc analysis was utilized. Statistical significance was defined as a P-value < 0.05. A detailed description of specific statistics is provided in Additional file 2: Table S2.

## Results

### SCI induces neuronal damage and glial cell activation

**Fig. 2:**
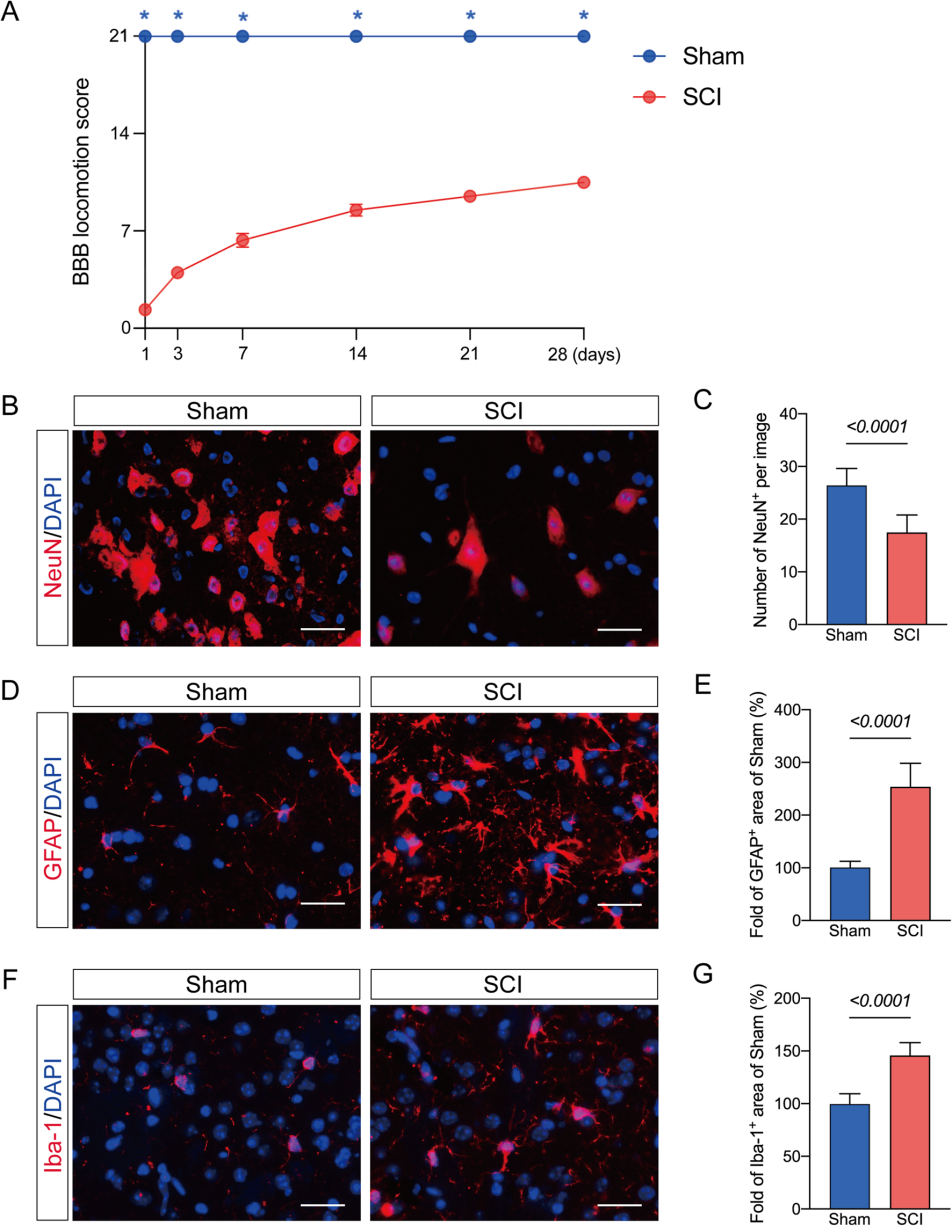
SCI induces neuronal damage and glial cell activation. **A** Changes of Basso Beattie Bresnahan (BBB) scale at 1, 3, 7, 14, 21 and 28 days after SCI. *P < 0.05. Data are presented as the mean ± SD (*n* = 6 per group) **B** Representative images of NeuN-positive cells staining in the Sham and SCI groups; scale bar = 25 μm. **C** Number of NeuN-positive cells of each group. **D** Representative images of GFAP-positive cells staining in the two groups; scale bar = 25 μm. **E** Quantitative analysis of GFAP-positive cells in the SCI group compared with the sham group **F** Representative images of Iba-1-positive cells staining in the two groups; scale bar = 25 μm. **G** Quantitative analysis of Iba1-positive cells in each group. Data are presented as the mean ± SD (*n* = 6 per group)

Following SCI, the BBB score was used to evaluate motor function on days 1, 3, 7, 14, 21 and 28. The BBB scores showed that the SCI group's motor function was considerably worse than the sham-operated group. On day three after SCI, the motor function of the hindlimb began to recover, the BBB scores began to rise, and the BBB score was highest on day 28 after SCI, with significant recovery of hindlimb function (Fig. 2A).

Neuronal loss and glia activation, including astrocytes and microglia, were reportedly involved in post-SCI pathological changes [14, 33]. In this current study, the number of neurons, astrocytes, and microglia was further assessed by immunofluorescence assay. At 28 days after SCI, mice in the SCI group demonstrated a significant decrease in NeuN-positive neurons and significant increases in GFAP and Iba-1 intensity than Sham mice (Fig. 2B–G).

### IL-18 in astrocytes is involved in the inflammatory response at the early stage after SCI

**Fig. 3:**
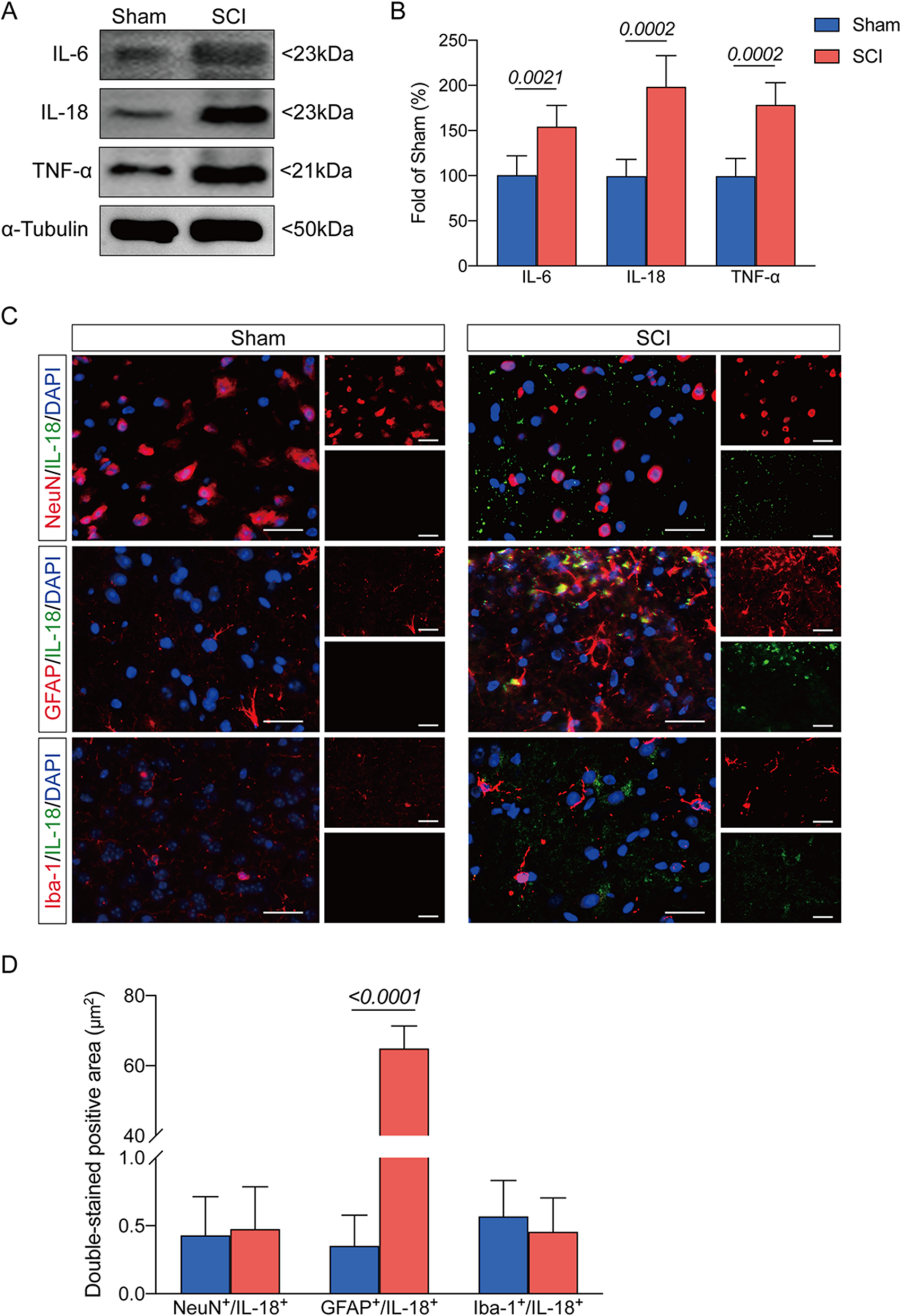
IL-18 in astrocytes is involved in the inflammatory response at the early stage after SCI. **A** Representative western blot images of inflammatory-associated proteins including IL-6, IL-18 and TNF-α in the Sham and SCI groups. **B** Ratio between the optical density value of IL-6, IL-18 and TNF-α in each group. **C** Representative images of IL-18- and NeuN-positive cells, IL-18- and GFAP-positive cells and IL-18- and Iba1-positive cells in the two groups; scale bar = 25 μm. **D** Double-stained positive area in the two groups. Data are presented as the mean ± SD (*n* = 6 per group)

Previous studies demonstrated that IL-18 induced inflammatory responses in motor dysfunction after SCI. The western blot results showed that mice in the SCI group had significantly higher expressions of IL-18, TNF-α, and IL-6 in the injured spinal cord than Sham group (Fig. 3A, B). Furthermore, the IL-18 colocalization with neurons, astrocytes, and microglia were evaluated by immunofluorescence assay. We found a remarkable elevation of the colocalization of IL-18 with GFAP in the spinal cord after SCI compared to the Sham group (Fig. 3C, D). Moreover, there was no obvious difference in the colocalization of IL-18 with NeuN or Iba-1 between mice in the SCI group and Sham group (Fig. 3C, D).These results indicated that IL-18 in astrocytes but not neurons or microglia is involved in the inflammatory response at the early stage after SCI.

### Depletion of NLPR3 signal pathway in the astrocyte ameliorates motor dysfunction post-SCI

The NLRP3–IL-18 activation in the astrocyte has been demonstrated to promote neurological dysfunction. Next, we aimed to investigate if the NLRP3–IL-18 signal played a role in astrogliosis development under the SCI state. In a previous study, conditional NLRP3 knockout mice bearing loxp-flanked NLRP3 alleles were crossed with mice expressing GFAP-Cre to create double transgenic animals with astrocytic-specific inactivation of NLRP3 (knockout [KO]; NLRP3-KO) (Additional file 3) [34]. Mice that were NLRP3-KO and wild type (WT) received additional SCI exposure treatment. At seven days after SCI, the NLRP3-KO mice group showed a considerable reduction in NLRP3 colocalization with GFAP.

**Fig. 4:**
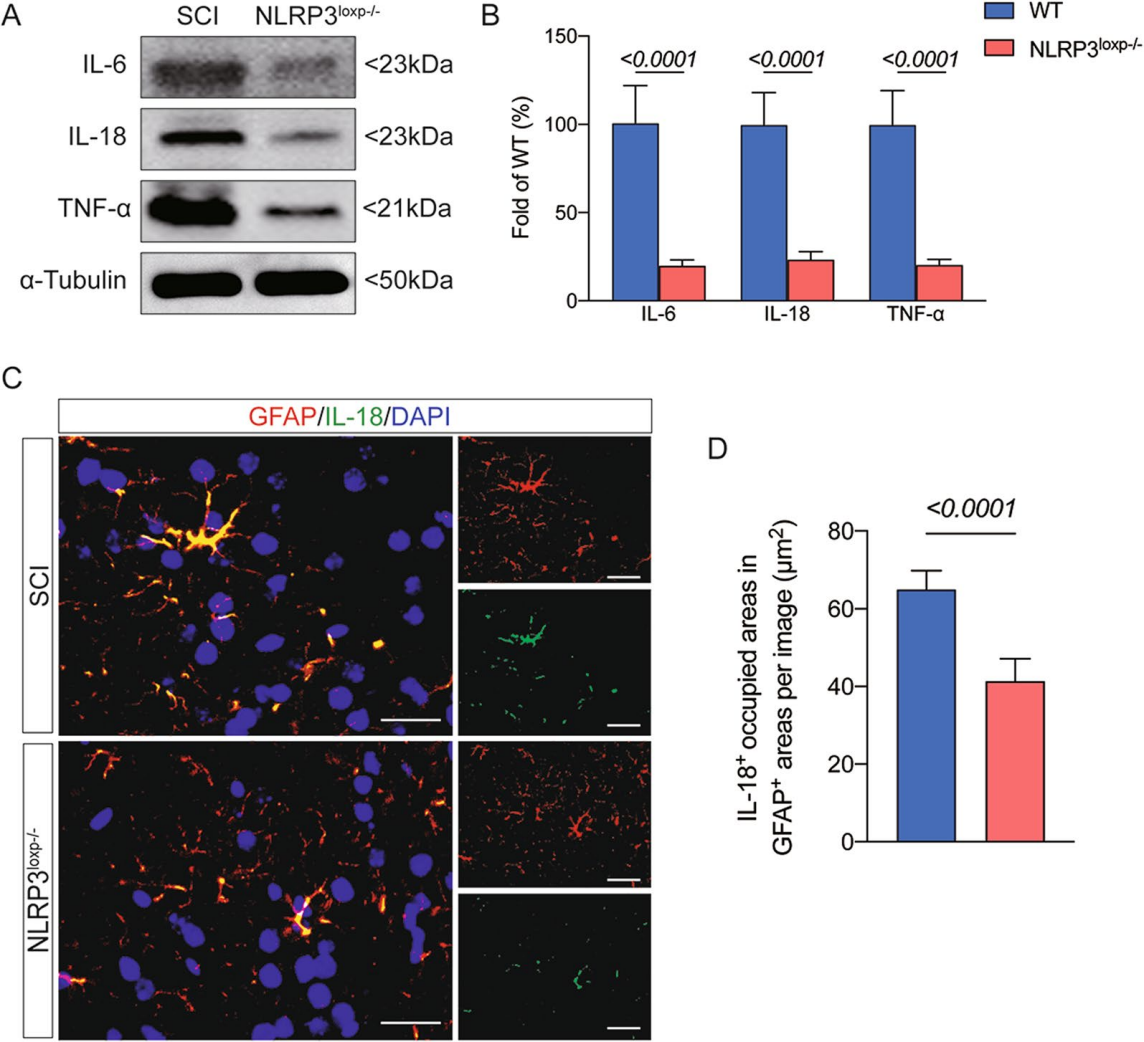
Depletion of NLPR3 signal pathway in the astrocyte ameliorates motor dysfunction post-SCI. **A** Representative western blot images of inflammatory-associated proteins including IL-6, IL-18 and TNF-α in the WT and NLRP3^loxp−/−^ + SCI groups. Data are presented as the mean ± SD (*n* = 6 per group). **B** Ratio between the optical density value of IL-6, IL-18 and TNF-α in each group. **C** Representative images of IL-18- and GFAP-positive cells in the two groups; scale bar = 25 μm. **D** IL-18^+^ occupied areas in GFAP^+^ areas in the two groups. Data are presented as the mean ± SD (*n* = 6 per group)

**Fig. 5:**
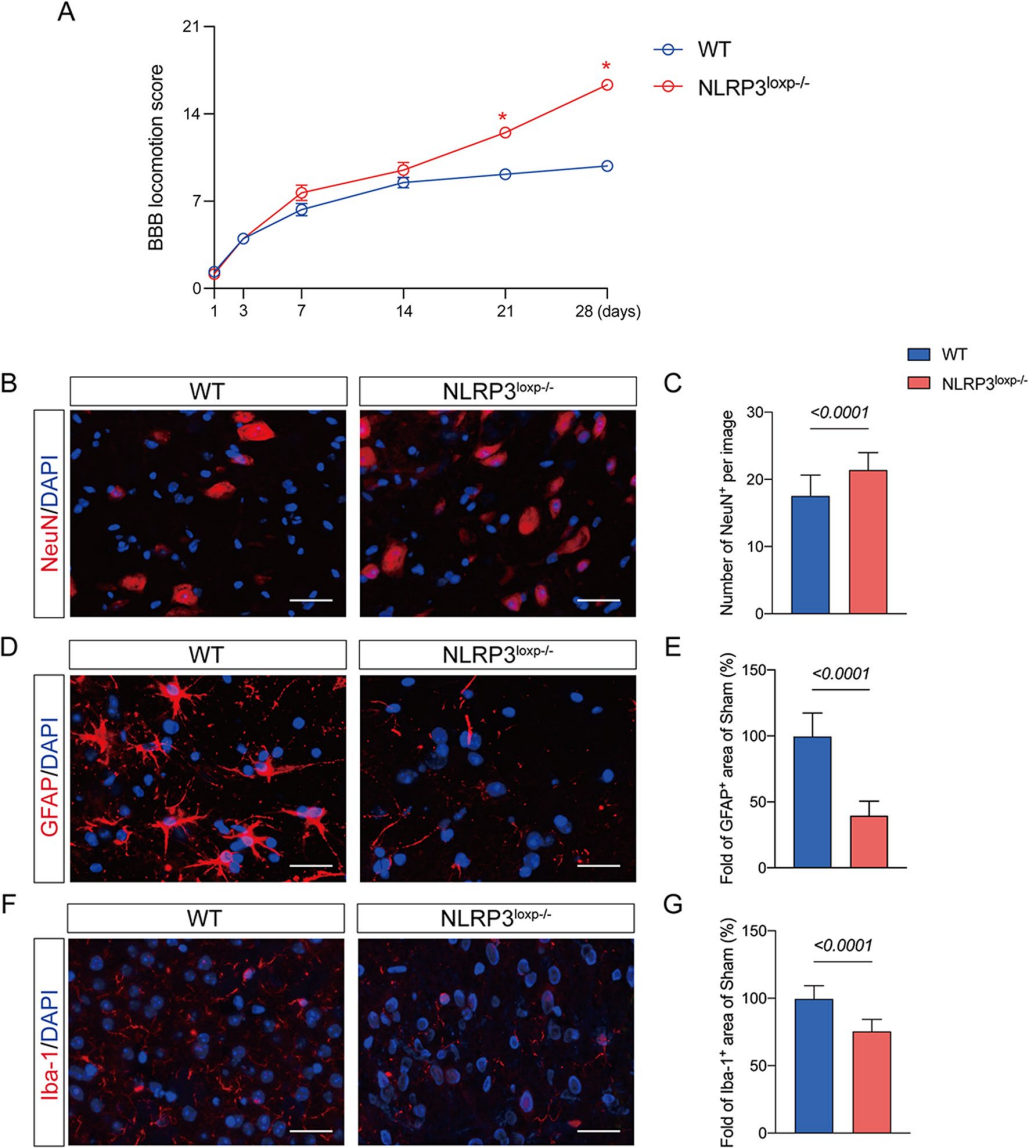
Depletion of NLPR3 signal pathway in the astrocyte ameliorates motor dysfunction post-SCI. **A** Changes of Basso Beattie Bresnahan (BBB) scale at 1, 3, 7, 14, 21 and 28 days after SCI in the WT group and NLRP3^loxp−/−^ group. *P < 0.05. Data are presented as the mean ± SD (*n* = 6 per group). **B** Representative images of NeuN-positive cells staining in the two groups; scale bar = 25 μm. **C** Number of NeuN-positive cells of each group. **D** Representative images of GFAP-positive cells staining in the two groups; scale bar = 25 μm. **E** Quantitative analysis of GFAP-positive cells in the NLRP3^loxp−/−^ group compared with the WT group **F** Representative images of Iba-1-positive cells staining in the two groups; scale bar = 25 μm. **G** Quantitative analysis of Iba1-positive cells in each group. Data are presented as the mean ± SD (*n* = 6 per group)

Moreover, it was also shown that the colocalization of IL-18 with GFAP was remarkably reduced in the mice with NLRP3-KO compared with WT mice after SCI (Fig. 4C, D). Furthermore, there were significant reductions of IL-6, IL-18 and TNF-α expressions in the injured spinal cord in NLRP3-KO mice than in WT mice post-SCI (Fig. 4A, B). Notably, NLRP3-KO mice exhibited increased BBB scores at 21 and 28 days after SCI compared with the WT mice group (Fig. 5A). Moreover, we found a remarkable increase of NeuN-positive cells and a decrease of GFAP and Iba-1 intensity in the mice with NLRP3-KO compared with WT mice after SCI at 28 days after SCI (Fig. 5B–G). These results revealed the NLRP3 signal's role in IL-18-induced astrogliosis and inflammatory response after SCI (Additional file 4).

### AM treatment ameliorates motor dysfunction post-SCI through NLRP3–IL-18 pathway

**Fig. 6:**
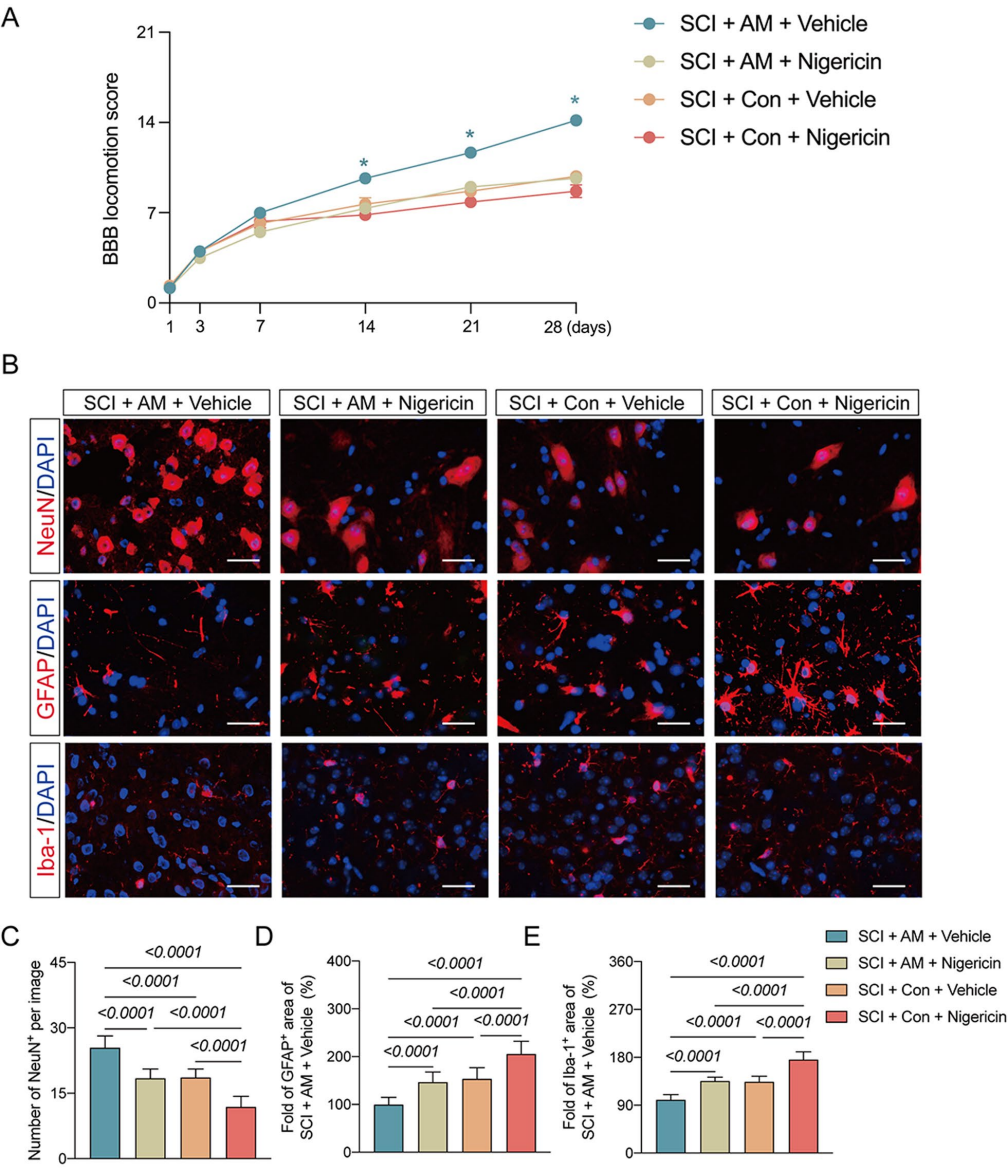
AM treatment ameliorates motor dysfunction post-SCI through NLRP3–IL-18 pathway. **A** Changes of Basso Beattie Bresnahan (BBB) scale at 1, 3, 7, 14, 21 and 28 day safter SCI in the SCI + AM + Vehicle, SCI + AM + Nigericin, SCI + Con + Vehicle and SCI + Con + Nigericin groups. *P < 0.05. Data are presented as the mean ± SD (*n* = 6 per group). **B** Representative images of NeuN-, GFAP-, and Iba1-positive cells staining in the four groups; scale bar = 25 μm. **B**–**E** Quantitative analysis of NeuN-, GFAP-, and Iba1-positive cells in each group. Data are presented as the mean ± SD (*n* = 6 per group)

**Fig. 7:**
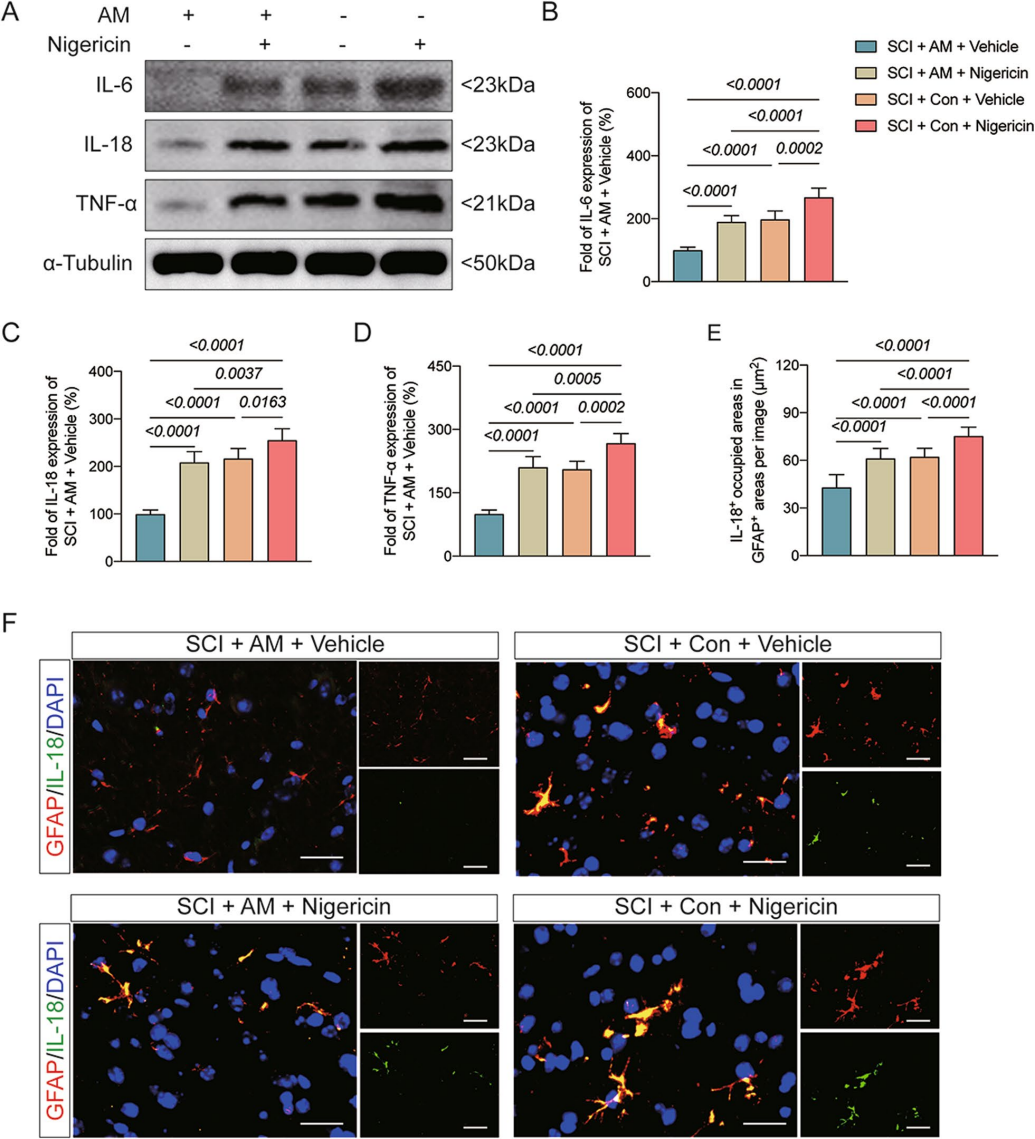
AM treatment ameliorates motor dysfunction post-SCI through NLRP3–IL-18 pathway. **A** Representative western blot images of inflammatory-associated proteins including IL-6, IL-18 and TNF-α in the SCI + AM + Vehicle, SCI + AM + Nigericin, SCI + Con + Vehicle and SCI + Con + Nigericin groups. Data are presented as the mean ± SD (*n* = 6 per group). **B**–**D** Ratio between the optical density value of IL-6, IL-18 and TNF-α in each group. **E** IL-18-positive cells occupied areas in GFAP-positive cells areas in the four groups. **F** Representative images of IL-18- and GFAP-positive cells in the four groups; scale bar = 25 μm. Data are presented as the mean ± SD (*n* = 6 per group)

At 14, 21 and 28 days after SCI, it was shown that the BBB scores were significantly higher in mice treated with SCI plus AM and vehicle administration than SCI plus control and vehicle administration (Fig. 6A). Furthermore, at 14, 21 and 28 days, BBB scores in mice from the SCI plus vehicle administration, and SCI plus nigericin administration groups did not significantly differ (Fig. 6A). At 28 days after SCI, we found that mice treated with SCI plus AM and vehicle administration exhibited increased NeuN-positive cells and decreased GFAP and Iba-1 intensity in the spinal cord than SCI plus control and vehicle administration (Fig. 6B–E). At seven days after SCI, it was shown that expressions in IL-18, IL-6, and TNF-α, and the colocalization of IL-18 with GFAP were significantly decreased in mice treated with SCI plus AM and vehicle administration than SCI plus control and vehicle administration (Fig. 7A–F). There were decreased NeuN-positive cells, increased GFAP and Iba-1 intensity in the spinal cord at 28 days, and increased expressions in IL-18, IL-6, and TNF-α, elevated the colocalization of IL-18 with GFAP at seven days after SCI in mice exposed to SCI plus AM and nigericin administration compared to SCI plus AM and vehicle administration (Fig. 6B–E, 7A–F). Moreover, all the index was further aggravated in mice exposed to SCI plus control and nigericin administration compared to SCI plus AM and vehicle administration (Fig. 6B–E, 7A–F). These data revealed that AM treatment ameliorates motor dysfunction post-SCI through NLRP3–IL-18 pathway.

## Discussion

Here, we provide compelling evidence that astrocyte-specific NLRP3 knockout significantly reversed SCI-induced motor dysfunction, decreased neuronal cells, activation of astrocytes and microglia, increased IL-18 colocalized with astrocytes, and aggravated inflammatory responses as indicated by IL-6 and TNF-α. Besides, AM treatment significantly reduced motor dysfunction, increased neuronal cells, and decreased the astrocytes and microglia activation, whereas an activator of NLRP3 nigericin significantly reversed these changes. The potential mechanism of AM against SCI-induced motor dysfunction may be associated with NLRP3–IL-18 signaling pathway.

**Fig. 8:**
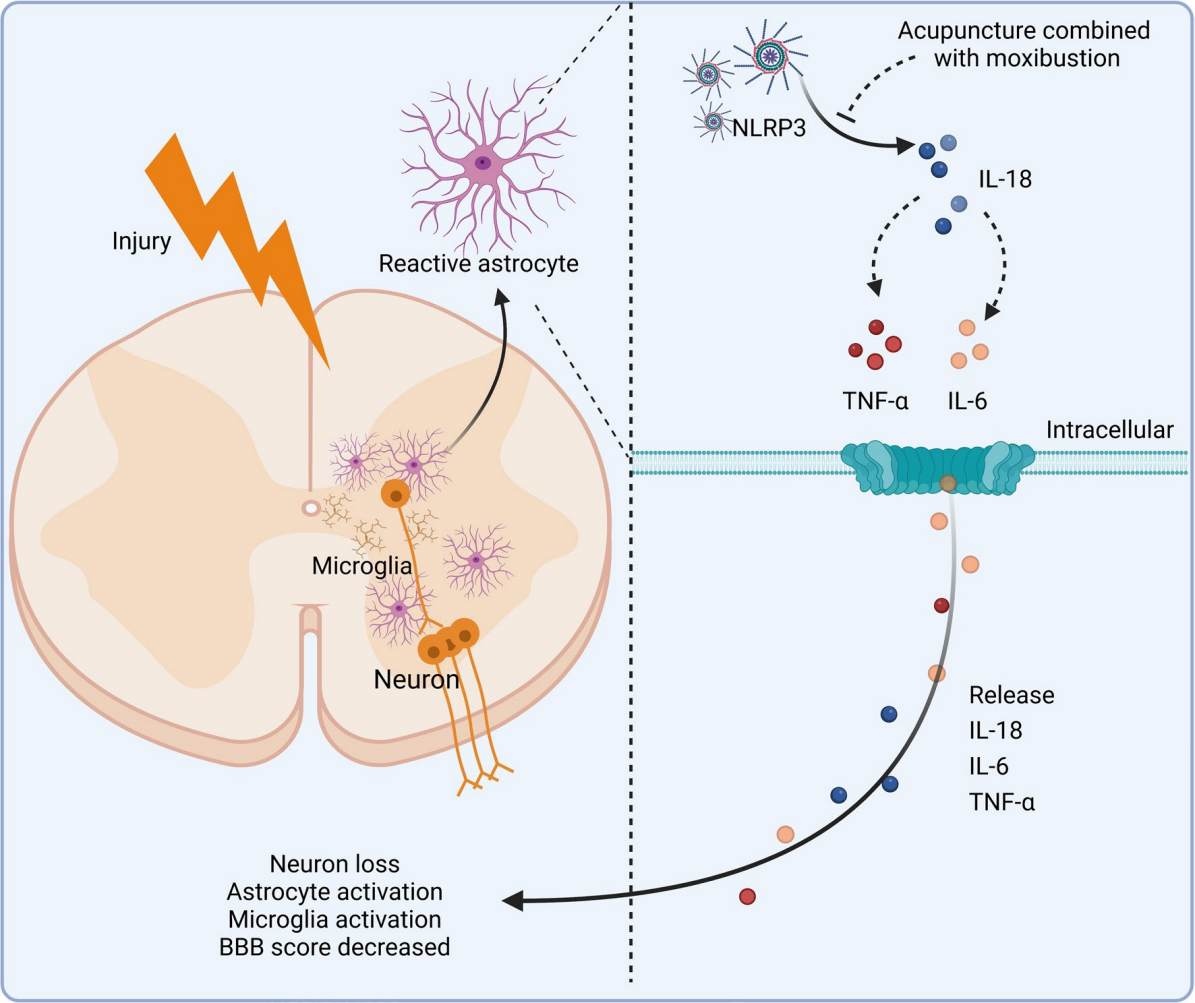
Schematic diagram of AM treatment to reduce neuroinflammatory response (Drawn by Biorender.com). AM therapy reduces the neuroinflammatory response by inhibiting the NLRP3-IL-18 signaling pathway, thereby suppressing motor dysfunction caused by spinal cord injury

Persistent and diffuse secondary damage following SCI, including further tissue damage and neurodegeneration, can lead to significant expansion of the injury site to higher segments and, ultimately, to neurological dysfunction [35, 36]. Growing evidence suggested that early inflammatory responses post-SCI participated in long-term neurological dysfunction [5, 37, 38]. In the current study, we discovered that SCI caused by impact methods not only resulted in motor dysfunction in mice but also led to neuronal loss and glial activation in the spinal cord, which is in line with earlier research [39, 40]. Furthermore, we also found aggravated inflammatory responses indicated by increased IL-18, IL-6, and TNF-α at the early stage after SCI. These results indicated that the early-stage suppression of the inflammatory response might be a potential therapy against SCI (Fig. 8).

In animal models of bacterial infections and traumatic stress, IL-18-mediated inflammation has mostly been explored. Previous research highlighted the IL-18 role in exaggerating the inflammatory burden and inducing tissue damage. However, blocking IL-18 has emerged as a promising therapeutic target for inflammatory response treatment. Among the multiple inflammasome complexes, NLRP3 is one of the most relevant key regulators of various inflammatory responses in CNS trauma [41]. The NLRP3–IL-18 signaling pathway leads to tissue damage through the astrocytes' pathological activation [42–44]. After SCI, reactive astrocytes aid in glial scarring development, inhibiting neuronal regeneration and ultimately preventing neurological recovery [15, 17]. In the current investigation, we discovered that colocalization of IL-18 with astrocytes was substantially higher than neuron and microglia post-SCI.

Interestingly, astrocyte-specific NLRP3 knockout heavily decreased the IL-18 colocalization with astrocytes, consistent with a previous study [34]. Notably, astrocyte-specific NLRP3 knockout inhibited the inflammatory response at the early stage and improved motor dysfunction at the late-stage post-SCI. These results revealed that NLRP3–IL-18 signaling pathway activation in astrocytes at the early stage involved motor dysfunction after SCI.

In recent years, AM and electroacupuncture have been used as alternative treatments for many diseases [18, 45, 46]. The pathological alteration of spinal cord injury can be explained by damage to the "Governor Vessel" according to the traditional Chinese medicine theory of Zang-fu organs and meridians [23]. Atrophy and disuse are brought on by the malnutrition of tendons and muscles caused by qi and blood blockage. Therefore, the first options for treating SCI are Governor Vessel acupoints. "Dazhui" (GV14) is the confluence of the three Yang meridians of the hands and feet with the Governor Vessel. To dredge the meridians, acupuncture stimulation in GV 14 can inspire and stimulate Yang-qi throughout the body [47]. As the junction of the Governor Vessel and Belt Vessel, "Mingmen" (GV4) is also an acupoint on Governor Vessel. It brings together the genuine Yin and genuine Yang, which are the source of Yuan-qi and the doorway to life. It can control channels, activate collaterals, tonify Yang, and strengthen the kidneys by stimulating GV4 [48]. "Jiaji" points are in the back and waist of the body. Acupuncture at Jiaji has been shown that could improve blood circulation and relieve edema after SCI, and protect and promote axonal regeneration [49]. In the sciatic nerve projection region, which is dominated by the L3-6 spinal segments, there are two clusters of acupoints known as "Zusanli" (ST36), which have been linked to spinal cord plasticity [50]. The "Ciliao" (BL32) is situated across from the second posterior sacral foramen, between the posterior superior iliac spine and the posterior median line. At Ciliao, acupuncture may aid muscular function and blood flow rehabilitation [18]. Our current data showed that AM treatment significantly inhibited NLRP3-induced increased colocalization of IL-18 with astrocytes.

Moreover, AM treatment alleviated inflammatory responses at the early stage, ameliorated motor dysfunction and neuronal loss, and reduced glial activation post-SCI. Besides, our study also showed that nigericin injection, an NLRP3 activator, notably reduced the neuroprotective benefits of AM. The current concentration of nigericin could reverse the therapeutic effect of AM without causing significant damage to the motor ability of mice, so BBB scores in mice from the SCI plus vehicle administration, and SCI plus nigericin administration groups did not significantly differ at 14, 21 and 28 days. In addition, MCC950, a selective NOD-like receptor protein-3 (NLRP3) inflammasome inhibitor, has been reported to reduce the inflammatory response and improve neurological outcomes in mice model of SCI [51, 52]. These data suggested that AM has the potential to attenuate traumatic stress-induced motor dysfunction by the NLRP3–IL18 signaling pathway inhibition.

Our present study demonstrated that AM treatment alleviated motor dysfunction post-SCI. The NLPR3–IL18 signaling pathway inhibition in the astrocyte at the early stage contributed to the neuroprotective effects of AM treatment post-SCI. Our study suggested that AM treatment may benefit SCI recovery, providing evidence for the clinical use of AM post-SCI.

## Supplementary Information

**Additional file 1. **Key resources, including antibodies, reagents, and software.

**Additional file 2. **A detailed description of the specific statistics.

**Additional file 3. **Gene knockout strategy.

**Additional file 4. **Original western blots.
