# Supplementary material for: Acupuncture combined with moxibustion mitigates spinal cord injury-induced motor dysfunction in mice by NLRP3-IL-18 signaling pathway inhibition
Source: J Orthop Surg Res. 2023 Jun 9;18:419. doi: 10.1186/s13018-023-03902-6 (PMC10257262; doi:10.1186/s13018-023-03902-6)
Supplement: Supplementary file 1 — Additional file 1. Key resources, including antibodies, reagents, and software. [file 13018_2023_3902_MOESM1_ESM.doc]

Supplemental Table 1

Key resources, including antibodies, reagents, and software.

| **Animal** | **Source** | |
| --- | --- | --- |
| C57BL/6 mice | Changsheng Biotechnology Co. LTD, China | |
| Astrocyte-specific NLRP3-KO mice | Cyagen Biotechnology Co. LTD, China | |
| **Product** | **Code** | **Manufacturer** |
| [NeuN Rabbit Monoclonal Antibody](https://www.beyotime.com/product/AF1072.htm) | [ab104224](https://www.abcam.cn/neun-antibody-1b7-neuronal-marker-ab104224.html) | Abcam, Cambridge, UK |
| [GFAP Mouse Monoclonal Antibody](https://www.beyotime.com/product/AF0156.htm) | AF0156 | Beyotime, Shanghai, China |
| Iba-1 Goat polyclonal Antibody | Ab5076 | Abcam, Cambridge, UK |
| [IL-18 Rabbit polyclonal Antibody](https://www.beyotime.com/product/AF1072.htm) | AF7266 | Beyotime, Shanghai, China |
| [NLRP3 Rabbit Monoclonal Antibody](https://www.beyotime.com/product/AF1072.htm) | AF2165 | Beyotime, Shanghai, China |
| [NLRP3 Rabbit Polyclonal Antibody](https://www.beyotime.com/product/AF1072.htm) | K004108P | Solarbio, Beijing, China |
| [IL-6 Mouse Monoclonal Antibody](https://www.beyotime.com/product/AF1072.htm) | AF0201 | Beyotime, Shanghai, China |
| [TNF-α Rabbit Polyclonal Antibody](https://www.beyotime.com/product/AF1072.htm) | AF8208 | Beyotime, Shanghai, China |
| DAPI | P0131-25ml | Beyotime, Shanghai, China |
| GAPDH Rabbit polyclonal Antibody | K106389P | Solarbio, Beijing, China |
| CyTM3-Conjugated Goat Anti-mouse  IgG | A0521 | Beyotime, Shanghai, China |
| Cy3-labeled Donkey Anti-Goat IgG  (H+L) | A0502 | Beyotime, Shanghai, China |
| HRP-labeled Goat Anti-Rabbit IgG  (H+L) | A0208 | Beyotime, Shanghai, China |
| HRP-labeled Goat Anti-Mouse IgG  (H+L) | A0216 | Beyotime, Shanghai, China |
| FITC-Conjugated goat anti-Rabbit IgG | A0562 | Beyotime, Shanghai, China |
| sevoflurane | H200701172 | SHANGHAI HENGRUI PHARMACEUTICAL CO.LTD, Shanghai,  China |
| Nigericin sodium salt | HY-100381 | Shanghai MedChemExpress Biotechnology Co. LTD, China |
| SDS-PAGE Protein Sample Loading  Buffer | P0295FT | Beyotime, Shanghai, China |
| BCA Protein Assay Kit | P0011 | Beyotime, Shanghai, China |
| Cell lysis buffer for Western and IP | P0013 | Beyotime, Shanghai, China |
| QuickBlock™ Blocking Buffer for  Western Blot | P0252FT | Beyotime, Shanghai, China |
| Improved Citrate Antigen Retrieval  Solution | P0083 | Beyotime, Shanghai, China |
| QuickBlock™ Blocking Buffer for  Immunol Staining | P0260 | Beyotime, Shanghai, China |
| Triton X-100 | T8200 | Solarbio, Beijing, China |
| Proteinase K (20mg/ml) | ST533 | Beyotime, Shanghai, China |
| BeyoECL Plus | P0018M | Beyotime, Shanghai, China |
| **Software** | **version** | **manufacturers** |
| Analysis software for open field test | XR-XZ301 | Shanghai Xinruan Software, Shanghai, |
| ImageJ Fiji | Version 2.0 | Media Cybernetics, Inc. USA |
| **Equipment** | **version** | **manufacturers** |
| Fluorescence microscope | MF31 | Mshot, Guangzhou, China |
| Stereoscope | SA-150 | Yuyan instruments, Shanghai, China |

| Streptavidin/SAlexa Fluor 488 | K0068R-AF488 | Solarbio, Beijing, China |
| --- | --- | --- |
| Streptavidin/SAlexa Fluor594 | K1068R-AF594 | Solarbio, Beijing, China |
| CyTM3-Conjugated Goat Anti-mouse  IgG | A0521 | Beyotime, Shanghai, China |
| Cy3-labeled Donkey Anti-Goat IgG  (H+L) | A0502 | Beyotime, Shanghai, China |
| HRP-labeled Goat Anti-Rabbit IgG  (H+L) | A0208 | Beyotime, Shanghai, China |
| FITC goat anti-Rabbit IgG | A0562 | Beyotime, Shanghai, China |
| sevoflurane | H200701172 | SHANGHAI HENGRUI PHARMACEUTICAL CO.LTD, Shanghai,  China |
| DMSO | ST038 | Beyotime, Shanghai, China |
| [Spautin-1](https://www.beyotime.com/product/SC5498-5mg.htm) | SC5498 | Beyotime, Shanghai, China |
| SDS-PAGE Protein Sample Loading  Buffer | P0295FT | Beyotime, Shanghai, China |
| BCA Protein Assay Kit | P0011 | Beyotime, Shanghai, China |
| Cell lysis buffer for Western and IP | P0013 | Beyotime, Shanghai, China |
| QuickBlock™ Blocking Buffer for  Western Blot | P0252FT | Beyotime, Shanghai, China |
| Improved Citrate Antigen Retrieval  Solution | P0083 | Beyotime, Shanghai, China |
| QuickBlock™ Blocking Buffer for  Immunol Staining | P0260 | Beyotime, Shanghai, China |
| Triton X-100 | T8200 | Solarbio, Beijing, China |
| Proteinase K (20mg/ml) | ST533 | Beyotime, Shanghai, China |
| BeyoECL Plus | P0018M | Beyotime, Shanghai, China |
| **Software** | **version** | **manufacturers** |
| Analysis software for open field test | XR-XZ301 | Shanghai Xinruan Software, Shanghai, |

|  |  | China |
| --- | --- | --- |
| Analysis software for elevated plus maze | XR-XZ301 | Shanghai Xinruan Software, Shanghai,  China |
| ImageJ Fiji | Version 2.0 | Media Cybernetics, Inc. USA |
| **Equipment** | **version** | **manufacturers** |
| Fluorescence microscope | MF31 | Mshot, Guangzhou, China |
| Stereoscope | SA-150 | Yuyan instruments, Shanghai, China |
