# Supplementary material for: Acupuncture combined with moxibustion mitigates spinal cord injury-induced motor dysfunction in mice by NLRP3-IL-18 signaling pathway inhibition
Source: J Orthop Surg Res. 2023 Jun 9;18:419. doi: 10.1186/s13018-023-03902-6 (PMC10257262; doi:10.1186/s13018-023-03902-6)
Supplement: Supplementary file 2 — Additional file 2. A detailed description of the specific statistics. [file 13018_2023_3902_MOESM2_ESM.docx]

**Supplement Table 2**

**A detailed description of the specific statistics.**

**Figure 1**

| BBB | | | | | | | | | |
| --- | --- | --- | --- | --- | --- | --- | --- | --- | --- |
| Shapiro-Wilk test | | sham | | SCI | |  | |  | |
| W | |  | | 0.9426 | |  | |  | |
| P value | |  | | 0.6803 | |  | |  | |
| Passed normality test (alpha=0.05)? | |  | | Yes | |  | |  | |
| P value summary | |  | | ns | |  | |  | |
|  | | | | | | | | | |
| ANOVA table | SS | | DF | | MS | | F (DFn, DFd) | | P value |
| Interaction | 185.2 | | 5 | | 37.05 | | F (5, 60) = 88.32 | | P<0.0001 |
| Row Factor | 185.2 | | 5 | | 37.05 | | F (5, 60) = 88.32 | | P<0.0001 |
| Column Factor | 3684 | | 1 | | 3684 | | F (1, 60) = 8782 | | P<0.0001 |
| Residual | 25.17 | | 60 | | 0.4194 | |  | |  |

| Number of NeuN^+^ per image | | | | |
| --- | --- | --- | --- | --- |
| Shapiro-Wilk test | sham | SCI |  |  |
| W | 0.9052 | 0.9232 |  |  |
| P value | 0.0278 | 0.0689 |  |  |
| Passed normality test (alpha=0.05)? | no | Yes |  |  |
| P value summary | * | ns |  |  |
| Mann Whitney test | | | | |
| P value | <0.0001 |  |  |  |
| Exact or approximate P value? | exact |  |  |  |
| P value summary | **** |  |  |  |
| Significantly different (P < 0.05)? | Yes |  |  |  |
| One- or two-tailed P value? | Two-tailed |  |  |  |
| Sum of ranks in column A, B | 868.5,307.5 |  |  |  |
| Mann-Whitney U | 7.500 |  |  |  |

| Fold of GFAP^+^ area of Sham | | | | |
| --- | --- | --- | --- | --- |
| Shapiro-Wilk test | sham | SCI |  |  |
| W | 0.9551 | 0.9593 |  |  |
| P value | 0.3485 | 0.4242 |  |  |
| Passed normality test (alpha=0.05)? | Yes | Yes |  |  |
| P value summary | ns | ns |  |  |
|  | | | | |
| P value | <0.0001 |  |  |  |
| P value summary | **** |  |  |  |
| Significantly different (P < 0.05)? | Yes |  |  |  |
| One- or two-tailed P value? | Two-tailed |  |  |  |
| t, df | t=16.04, df=46 |  |  |  |

| Fold of Iba1^+^ area of Sham | | | | |
| --- | --- | --- | --- | --- |
| Shapiro-Wilk test | sham | SCI |  |  |
| W | 0.9466 | 0.9260 |  |  |
| P value | 0.2285 | 0.0793 |  |  |
| Passed normality test (alpha=0.05)? | Yes | Yes |  |  |
| P value summary | ns | ns |  |  |
|  | | | | |
| P value | <0.0001 |  |  |  |
| P value summary | **** |  |  |  |
| Significantly different (P < 0.05)? | Yes |  |  |  |
| One- or two-tailed P value? | Two-tailed |  |  |  |
| t, df | t=14.28, df=46 |  |  |  |

**Figure 2**

| Ratio of the optical density value of IL-6 (%sham) | | | | |
| --- | --- | --- | --- | --- |
| Shapiro-Wilk test | sham | SCI |  |  |
| W | 0.8346 | 0.9152 |  |  |
| P value | 0.1175 | 0.4715 |  |  |
| Passed normality test (alpha=0.05)? | Yes | Yes |  |  |
| P value summary | ns | ns |  |  |
|  | | | | |
| P value | 0.0020 |  |  |  |
| P value summary | ** |  |  |  |
| Significantly different (P < 0.05)? | Yes |  |  |  |
| One- or two-tailed P value? | Two-tailed |  |  |  |
| t, df | t=4.134, df=10 |  |  |  |

| Ratio of the optical density value of IL-18 (%sham) | | | | |
| --- | --- | --- | --- | --- |
| Shapiro-Wilk test | sham | SCI |  |  |
| W | 0.7982 | 0.9583 |  |  |
| P value | 0.0566 | 0.8066 |  |  |
| Passed normality test (alpha=0.05)? | Yes | Yes |  |  |
| P value summary | ns | ns |  |  |
|  | | | | |
| P value | 0.0001 |  |  |  |
| P value summary | *** |  |  |  |
| Significantly different (P < 0.05)? | yes |  |  |  |
| One- or two-tailed P value? | Two-tailed |  |  |  |
| t, df | t=6.189, df=10 |  |  |  |

| Ratio of the optical density value of TNF-α (%sham) | | | | |
| --- | --- | --- | --- | --- |
| Shapiro-Wilk test | sham | SCI |  |  |
| W | 0.9016 | 0.9780 |  |  |
| P value | 0.3835 | 0.9409 |  |  |
| Passed normality test (alpha=0.05)? | Yes | Yes |  |  |
| P value summary | ns | ns |  |  |
|  | | | | |
| P value | 0.0001 |  |  |  |
| P value summary | *** |  |  |  |
| Significantly different (P < 0.05)? | Yes |  |  |  |
| One- or two-tailed P value? | Two-tailed |  |  |  |
| t, df | t=6.147, df=10 |  |  |  |

| NeuN^+^/IL18^+^ double-stained positive area (μm^2^) | | | | |
| --- | --- | --- | --- | --- |
| Shapiro-Wilk test | sham | SCI |  |  |
| W | 0.9523 | 0.9284 |  |  |
| P value | 0.3035 | 0.0898 |  |  |
| Passed normality test (alpha=0.05)? | Yes | Yes |  |  |
| P value summary | ns | ns |  |  |
|  | | | | |
| P value | 0.5832 |  |  |  |
| P value summary | ns |  |  |  |
| Significantly different (P < 0.05)? | no |  |  |  |
| One- or two-tailed P value? | Two-tailed |  |  |  |
| t, df | t=0.5526, df=46 |  |  |  |

| GFAP^+^/IL18^+^ double-stained positive area (μm^2^) | | | | |
| --- | --- | --- | --- | --- |
| Shapiro-Wilk test | sham | SCI |  |  |
| W | 0.9254 | 0.9371 |  |  |
| P value | 0.0771 | 0.1405 |  |  |
| Passed normality test (alpha=0.05)? | Yes | Yes |  |  |
| P value summary | ns | ns |  |  |
|  | | | | |
| P value | <0.0001 |  |  |  |
| P value summary | **** |  |  |  |
| Significantly different (P < 0.05)? | Yes |  |  |  |
| One- or two-tailed P value? | Two-tailed |  |  |  |
| t, df | t=48.89, df=46 |  |  |  |

| Iba1^+^/IL18^+^ double-stained positive area (μm^2^) | | | | |
| --- | --- | --- | --- | --- |
| Shapiro-Wilk test | Sham | SCI |  |  |
| W | 0.9528 | 0.9360 |  |  |
| P value | 0.3106 | 0.1325 |  |  |
| Passed normality test (alpha=0.05)? | Yes | Yes |  |  |
| P value summary | ns | ns |  |  |
|  | | | | |
| P value | 0.1358 |  |  |  |
| P value summary | ns |  |  |  |
| Significantly different (P < 0.05)? | No |  |  |  |
| One- or two-tailed P value? | Two-tailed |  |  |  |
| t, df | t=1.58, df=46 |  |  |  |

**Figure 3**

| Ratio of the optical density value of IL-6 (%WT) | | | | |
| --- | --- | --- | --- | --- |
| Shapiro-Wilk test | WT | NLRP3^loxp-/-^ |  |  |
| W | 0.8346 | 0.9590 |  |  |
| P value | 0.1175 | 0.8120 |  |  |
| Passed normality test (alpha=0.05)? | Yes | Yes |  |  |
| P value summary | ns | ns |  |  |
|  | | | | |
| P value | <0.0001 |  |  |  |
| P value summary | **** |  |  |  |
| Significantly different (P < 0.05)? | Yes |  |  |  |
| One- or two-tailed P value? | Two-tailed |  |  |  |
| t, df | t=9.077, df=10 |  |  |  |

| Ratio of the optical density value of IL-18 (%WT) | | | | |
| --- | --- | --- | --- | --- |
| Shapiro-Wilk test | WT | NLRP3^loxp-/-^ |  |  |
| W | 0.7982 | 0.8312 |  |  |
| P value | 0.0566 | 0.1100 |  |  |
| Passed normality test (alpha=0.05)? | Yes | Yes |  |  |
| P value summary | ns | ns |  |  |
|  | | | | |
| P value | <0.0001 |  |  |  |
| P value summary | **** |  |  |  |
| Significantly different (P < 0.05)? | Yes |  |  |  |
| One- or two-tailed P value? | Two-tailed |  |  |  |
| t, df | t=9.817, df=10 |  |  |  |

| Ratio of the optical density value of TNF-α (%sham) | | | | |
| --- | --- | --- | --- | --- |
| Shapiro-Wilk test | WT | NLRP3^loxp-/-^ |  |  |
| W | 0.9016 | 0.8739 |  |  |
| P value | 0.3835 | 0.2423 |  |  |
| Passed normality test (alpha=0.05)? | Yes | Yes |  |  |
| P value summary | ns | ns |  |  |
|  | | | | |
| P value | <0.0001 |  |  |  |
| P value summary | **** |  |  |  |
| Significantly different (P < 0.05)? | Yes |  |  |  |
| One- or two-tailed P value? | Two-tailed |  |  |  |
| t, df | t=9.790, df=10 |  |  |  |

| IL18^+^ occupied areas in GFAP^+^ areas (μm^2^) | | | | |
| --- | --- | --- | --- | --- |
| Shapiro-Wilk test | WT | NLRP3^loxp-/-^ |  |  |
| W | 0.9353 | 0.9371 |  |  |
| P value | 0.1284 | 0.1404 |  |  |
| Passed normality test (alpha=0.05)? | Yes | Yes |  |  |
| P value summary | ns | ns |  |  |
|  | | | | |
| P value | <0.0001 |  |  |  |
| P value summary | **** |  |  |  |
| Significantly different (P < 0.05)? | Yes |  |  |  |
| One- or two-tailed P value? | Two-tailed |  |  |  |
| t, df | t=15.26, df=46 |  |  |  |

**Figure 4**

| BBB | | | | | | | | | |
| --- | --- | --- | --- | --- | --- | --- | --- | --- | --- |
| Shapiro-Wilk test | | WT | | NLRP3^loxp-/-^ | |  | |  | |
| W | | 0.8371 | | 0.9555 | |  | |  | |
| P value | | 0.1233 | | 0.7847 | |  | |  | |
| Passed normality test (alpha=0.05)? | | Yes | | Yes | |  | |  | |
| P value summary | | ns | | ns | |  | |  | |
|  | | | | | | | | | |
| ANOVA table | SS | | DF | | MS | | F (DFn, DFd) | | P value |
| Interaction | 96.50 | | 5 | | 19.30 | | F (5, 60) = 19.85 | | P<0.0001 |
| Row Factor | 1153 | | 5 | | 230.6 | | F (5, 60) = 237.2 | | P<0.0001 |
| Column Factor | 72.00 | | 1 | | 72.00 | | F (1, 60) = 74.06 | | P<0.0001 |
| Residual | 58.33 | | 60 | | 0.9722 | |  | |  |

| Number of NeuN-positive cells of each group | | | | |
| --- | --- | --- | --- | --- |
| Shapiro-Wilk test | WT | NLRP3^loxp-/-^ |  |  |
| W | 0.9408 | 0.9210 |  |  |
| P value | 0.1704 | 0.0615 |  |  |
| Passed normality test (alpha=0.05)? | Yes | Yes |  |  |
| P value summary | ns | ns |  |  |
|  | | | | |
| P value | <0.0001 |  |  |  |
| P value summary | **** |  |  |  |
| Significantly different (P < 0.05)? | Yes |  |  |  |
| One- or two-tailed P value? | Two-tailed |  |  |  |
| t, df | t=4.647, df=46 |  |  |  |

| Fold of GFAP^+^ area of Sham | | | | |
| --- | --- | --- | --- | --- |
| Shapiro-Wilk test | WT | NLRP3^loxp-/-^ |  |  |
| W | 0.9253 | 0.9438 |  |  |
| P value | 0.0768 | 0.1985 |  |  |
| Passed normality test (alpha=0.05)? | Yes | Yes |  |  |
| P value summary | ns | ns |  |  |
|  | | | | |
| P value | <0.0001 |  |  |  |
| P value summary | **** |  |  |  |
| Significantly different (P < 0.05)? | Yes |  |  |  |
| One- or two-tailed P value? | Two-tailed |  |  |  |
| t, df | t=14.03, df=46 |  |  |  |

| Fold of Iba1^+^ area of Sham | | | | |
| --- | --- | --- | --- | --- |
| Shapiro-Wilk test | WT | NLRP3^loxp-/-^ |  |  |
| W | 0.9417 | 0.9679 |  |  |
| P value | 0.1776 | 0.6147 |  |  |
| Passed normality test (alpha=0.05)? | Yes | Yes |  |  |
| P value summary | ns | ns |  |  |
|  | | | | |
| P value | <0.0001 |  |  |  |
| P value summary | **** |  |  |  |
| Significantly different (P < 0.05)? | Yes |  |  |  |
| One- or two-tailed P value? | Two-tailed |  |  |  |
| t, df | t=8.910, df=46 |  |  |  |

**Figure 5**

| BBB | | | | | |
| --- | --- | --- | --- | --- | --- |
| Shapiro-Wilk test | SCI + AM + Vehicle | SCI + AM + Nigericin | SCI + Con + Vehicle | SCI + Con +  Nigericin | |
| W | 0.9445 | 0.9307 | 0.9821 | 0.9744 | |
| P value | 0.2050 | 0.1011 | 0.9313 | 0.7748 | |
| Passed normality test (alpha=0.05)? | Yes | Yes | Yes | Yes | |
| P value summary | ns | ns | ns | ns | |
|  | | | | | |
| ANOVA table | SS | DF | MS | F (DFn, DFd) | P value |
| Interaction | 92.02 | 15 | 6.135 | F (15, 120) = 9.183 | P<0.0001 |
| Row Factor | 1461 | 5 | 292.2 | F (5, 120) = 437.4 | P<0.0001 |
| Column Factor | 100.9 | 3 | 33.62 | F (3, 120) = 50.32 | P<0.0001 |
| Residual | 80.17 | 120 | 0.6681 |  |  |
|  | | | | | |
| Tukey's multiple comparisons test | Mean Diff. | 95.00% CI of diff. | Significant? | Summary | |
| SCI + AM + Vehicle vs. SCI + AM + Nigericin | -21.8830 | -26.9453 to -16.8206 | Yes | **** | |
| SCI + AM + Vehicle vs. SCI + Con + Vehicle | -21.7850 | -26.3301 to -17.2400 | Yes | **** | |
| SCI + AM + Vehicle vs. SCI + Con + Nigericin | -34.9124 | -38.1820 to -31.6427 | Yes | **** | |
| SCI + AM + Nigericin vs. SCI + Con + Vehicle | 0.0979167 | -4.34852 to 4.54435 | No | ns | |
| SCI + AM + Nigericin vs. SCI + Con + Nigericin | -13.0294 | -18.2357 to -7.82315 | Yes | **** | |
| SCI + Con + Vehicle vs. SCI + Con + Nigericin | -13.1273 | -18.1019 to -8.15276 | Yes | **** | |

| Number of NeuN-positive cells of each group | | | | |
| --- | --- | --- | --- | --- |
| Shapiro-Wilk test | SCI + AM + Vehicle | SCI + AM + Nigericin | SCI + Con + Vehicle | SCI + Con +  Nigericin |
| W | 0.9234 | 0.9311 | 0.9562 | 0.9387 |
| P value | 0.0696 | 0.1033 | 0.3675 | 0.1529 |
| Passed normality test (alpha=0.05)? | Yes | Yes | Yes | Yes |
| P value summary | ns | ns | ns | ns |
|  | | | | |
| ANOVA table | SS | DF | MS | F (DFn, DFd) |
| Treatment (between columns) | 2215.11 | 3 | 738.372 | F (3, 92) = 137.499 |
| Residual (within columns) | 494.042 | 92 | 5.37002 |  |
| Total | 2709.16 | 95 |  |  |
|  | | | | |
| Tukey's multiple comparisons test | Mean Diff. | 95.00% CI of diff. | Significant? | Summary |
| SCI + AM + Vehicle vs. SCI + AM + Nigericin | 7.04167 | 5.29127 to 8.79207 | Yes | **** |
| SCI + AM + Vehicle vs. SCI + Con + Vehicle | 6.83333 | 5.08293 to 8.58373 | Yes | **** |
| SCI + AM + Vehicle vs. SCI + Con + Nigericin | 13.5833 | 11.8329 to 15.3337 | Yes | **** |
| SCI + AM + Nigericin vs. SCI + Con + Vehicle | -0.208333 | -1.95873 to 1.54207 | No | ns |
| SCI + AM + Nigericin vs. SCI + Con + Nigericin | 6.54167 | 4.79127 to 8.29207 | Yes | **** |
| SCI + Con + Vehicle vs. SCI + Con + Nigericin | 6.75000 | 4.99960 to 8.50040 | Yes | **** |

| Fold of GFAP^+^ area of SCI + AM + Vehicle (%) | | | | |
| --- | --- | --- | --- | --- |
| Shapiro-Wilk test | SCI + AM + Vehicle | SCI + AM + Nigericin | SCI + Con + Vehicle | SCI + Con +  Nigericin |
| W | 0.9365 | 0.9367 | 0.9776 | 0.9357 |
| P value | 0.1360 | 0.1376 | 0.8470 | 0.1308 |
| Passed normality test (alpha=0.05)? | Yes | Yes | Yes | Yes |
| P value summary | ns | ns | ns | ns |
|  | | | | |
| ANOVA table | SS | DF | MS | F (DFn, DFd) |
| Treatment (between columns) | 135696 | 3 | 45232.0 | F (3, 92) = 92.7959 |
| Residual (within columns) | 44844.0 | 92 | 487.435 |  |
| Total | 180540 | 95 |  |  |
|  | | | | |
| Tukey's multiple comparisons test | Mean Diff. | 95.00% CI of diff. | Significant? | Summary |
| SCI + AM + Vehicle vs. SCI + AM + Nigericin | -46.9500 | -63.6266 to -30.2734 | Yes | **** |
| SCI + AM + Vehicle vs. SCI + Con + Vehicle | -53.8500 | -70.5266 to -37.1734 | Yes | **** |
| SCI + AM + Vehicle vs. SCI + Con + Nigericin | -106.050 | -122.727 to -89.3734 | Yes | **** |
| SCI + AM + Nigericin vs. SCI + Con + Vehicle | -6.90000 | -23.5766 to 9.77660 | No | ns |
| SCI + AM + Nigericin vs. SCI + Con + Nigericin | -59.1000 | -75.7766 to -42.4234 | Yes | **** |
| SCI + Con + Vehicle vs. SCI + Con + Nigericin | -52.2000 | -68.8766 to -35.5234 | Yes | **** |

| Fold of Iba1^+^ area of SCI + AM + Vehicle (%) | | | | |
| --- | --- | --- | --- | --- |
| Shapiro-Wilk test | SCI + AM + Vehicle | SCI + AM + Nigericin | SCI + Con + Vehicle | SCI + Con +  Nigericin |
| W | 0.9716 | 0.9363 | 0.9634 | 0.9624 |
| P value | 0.7054 | 0.1352 | 0.5113 | 0.4885 |
| Passed normality test (alpha=0.05)? | Yes | Yes | Yes | Yes |
| P value summary | ns | ns | ns | ns |
|  | | | | |
| ANOVA table | SS | DF | MS | F (DFn, DFd) |
| Treatment (between columns) | 68650.4 | 3 | 22883.5 | F (3, 92) = 190.558 |
| Residual (within columns) | 11048.0 | 92 | 120.087 |  |
| Total | 79698.3 | 95 |  |  |
|  | | | | |
| Tukey's multiple comparisons test | Mean Diff. | 95.00% CI of diff. | Significant? | Summary |
| SCI + AM + Vehicle vs. SCI + AM + Nigericin | -35.1046 | -43.3820 to -26.8271 | Yes | **** |
| SCI + AM + Vehicle vs. SCI + Con + Vehicle | -33.3542 | -41.6316 to -25.0767 | Yes | **** |
| SCI + AM + Vehicle vs. SCI + Con + Nigericin | -75.4542 | -83.7316 to -67.1767 | Yes | **** |
| SCI + AM + Nigericin vs. SCI + Con + Vehicle | 1.75042 | -6.52703 to 10.0279 | No | ns |
| SCI + AM + Nigericin vs. SCI + Con + Nigericin | -40.3496 | -48.6270 to -32.0721 | Yes | **** |
| SCI + Con + Vehicle vs. SCI + Con + Nigericin | -42.1000 | -50.3774 to -33.8226 | Yes | **** |

**Figure 6**

| Ratio of the optical density value of IL-6 (%SCI + AM + Vehicle) | | | | |
| --- | --- | --- | --- | --- |
| Shapiro-Wilk test | SCI + AM + Vehicle | SCI + AM + Nigericin | SCI + Con + Vehicle | SCI + Con +  Nigericin |
| W | 0.8424 | 0.9337 | 0.8587 | 0.9203 |
| P value | 0.1364 | 0.6088 | 0.1847 | 0.5075 |
| Passed normality test (alpha=0.05)? | Yes | Yes | Yes | Yes |
| P value summary | ns | ns | ns | ns |
|  | | | | |
| ANOVA table | SS | DF | MS | F (DFn, DFd) |
| Treatment (between columns) | 85799.8 | 3 | 28599.9 | F (3, 20) = 53.2570 |
| Residual (within columns) | 10740.3 | 20 | 537.017 |  |
| Total | 96540.1 | 23 |  |  |
|  | | | | |
| Tukey's multiple comparisons test | Mean Diff. | 95.00% CI of diff. | Significant? | Summary |
| SCI + AM + Vehicle vs. SCI + AM + Nigericin | -90.0500 | -127.498 to -52.6022 | No | **** |
| SCI + AM + Vehicle vs. SCI + Con + Vehicle | -98.0267 | -135.474 to -60.5788 | Yes | **** |
| SCI + AM + Vehicle vs. SCI + Con + Nigericin | -168.350 | -205.798 to -130.902 | Yes | **** |
| SCI + AM + Nigericin vs. SCI + Con + Vehicle | -7.97667 | -45.4245 to 29.4712 | No | ns |
| SCI + AM + Nigericin vs. SCI + Con + Nigericin | -78.3000 | -115.748 to -40.8522 | Yes | **** |
| SCI + Con + Vehicle vs. SCI + Con + Nigericin | -70.3233 | -107.771 to -32.8755 | Yes | **** |

| Ratio of the optical density value of IL-18 (%SCI + AM + Vehicle) | | | | |
| --- | --- | --- | --- | --- |
| Shapiro-Wilk test | SCI + AM + Vehicle | SCI + AM + Nigericin | SCI + Con + Vehicle | SCI + Con +  Nigericin |
| W | 0.8868 | 0.8945 | 0.8655 | 0.8426 |
| P value | 0.3020 | 0.3427 | 0.2090 | 0.1369 |
| Passed normality test (alpha=0.05)? | Yes | Yes | Yes | Yes |
| P value summary | ns | ns | ns | ns |
|  | | | | |
| ANOVA table | SS | DF | MS | F (DFn, DFd) |
| Treatment (between columns) | 80296.8 | 3 | 26765.6 | F (3, 20) = 65.4595 |
| Residual (within columns) | 8177.76 | 20 | 408.888 |  |
| Total | 88474.6 | 23 |  |  |
|  | | | | |
| Tukey's multiple comparisons test | Mean Diff. | 95.00% CI of diff. | Significant? | Summary |
| SCI + AM + Vehicle vs. SCI + AM + Nigericin | -109.152 | -141.828 to -76.4752 | Yes | **** |
| SCI + AM + Vehicle vs. SCI + Con + Vehicle | -116.835 | -149.511 to  -84.1586 | Yes | **** |
| SCI + AM + Vehicle vs. SCI + Con + Nigericin | -155.675 | -188.351 to -122.999 | Yes | **** |
| SCI + AM + Nigericin vs. SCI + Con + Vehicle | -7.68333 | -40.3598 to 24.9931 | No | ns |
| SCI + AM + Nigericin vs. SCI + Con + Nigericin | -46.5233 | -79.1998 to -13.8469 | Yes | ** |
| SCI + Con + Vehicle vs. SCI + Con + Nigericin | -38.8400 | -71.5164 to -6.16358 | Yes | * |

| Ratio of the optical density value of TNF-α (%SCI + AM + Vehicle) | | | | |
| --- | --- | --- | --- | --- |
| Shapiro-Wilk test | SCI + AM + Vehicle | SCI + AM + Nigericin | SCI + Con + Vehicle | SCI + Con +  Nigericin |
| W | 0.9502 | 0.8202 | 0.8219 | 0.8381 |
| P value | 0.7422 | 0.0885 | 0.0917 | 0.1257 |
| Passed normality test (alpha=0.05)? | Yes | Yes | Yes | Yes |
| P value summary | ns | ns | ns | ns |
|  | | | | |
| ANOVA table | SS | DF | MS | F (DFn, DFd) |
| Treatment (between columns) | 88355.0 | 3 | 29451.7 | F (3, 20) = 72.2853 |
| Residual (within columns) | 8148.74 | 20 | 407.437 |  |
| Total | 96503.8 | 23 |  |  |
|  | | | | |
| Tukey's multiple comparisons test | Mean Diff. | 95.00% CI of diff. | Significant? | Summary |
| SCI + AM + Vehicle vs. SCI + AM + Nigericin | -111.045 | -143.663 to -78.4266 | Yes | **** |
| SCI + AM + Vehicle vs. SCI + Con + Vehicle | -106.000 | -138.618 to -73.3816 | Yes | **** |
| SCI + AM + Vehicle vs. SCI + Con + Nigericin | -167.998 | -200.617 to -135.380 | Yes | **** |
| SCI + AM + Nigericin vs. SCI + Con + Vehicle | 5.04500 | -27.5734 to 37.6634 | No | ns |
| SCI + AM + Nigericin vs. SCI + Con + Nigericin | -56.9533 | -89.5717 to -24.3349 | Yes | *** |
| SCI + Con + Vehicle vs. SCI + Con + Nigericin | -61.9983 | -94.6167 to -29.3799 | Yes | *** |

| IL18^+^ occupied areas in GFAP^+^ areas (μm^2^) | | | | |
| --- | --- | --- | --- | --- |
| Shapiro-Wilk test | SCI + AM + Vehicle | SCI + AM + Nigericin | SCI + Con + Vehicle | SCI + Con +  Nigericin |
| W | 0.9445 | 0.9307 | 0.9821 | 0.9744 |
| P value | 0.2050 | 0.1011 | 0.9313 | 0.7748 |
| Passed normality test (alpha=0.05)? | Yes | Yes | Yes | Yes |
| P value summary | ns | ns | ns | ns |
|  | | | | |
| ANOVA table | SS | DF | MS | F (DFn, DFd) |
| Treatment (between columns) | 15086.6 | 3 | 5028.86 | F (2.46866, 56.7792) = 149.733 |
| Residual (within columns) | 414.806 | 23 | 18.0351 | F (23, 69) = 0.536991 |
| Total | 2317.39 | 69 | 33.5854 |  |
|  | | | | |
| Tukey's multiple comparisons test | Mean Diff. | 95.00% CI of diff. | Significant? | Summary |
| SCI + AM + Vehicle vs. SCI + AM + Nigericin | -21.8830 | -26.9453 to -16.8206 | Yes | **** |
| SCI + AM + Vehicle vs. SCI + Con + Vehicle | -21.7850 | -26.3301 to -17.2400 | Yes | **** |
| SCI + AM + Vehicle vs. SCI + Con + Nigericin | -34.9124 | -38.1820 to -31.6427 | Yes | **** |
| SCI + AM + Nigericin vs. SCI + Con + Vehicle | 0.0979167 | -4.34852 to 4.54435 | No | ns |
| SCI + AM + Nigericin vs. SCI + Con + Nigericin | -13.0294 | -18.2357 to -7.82315 | Yes | **** |
| SCI + Con + Vehicle vs. SCI + Con + Nigericin | -13.1273 | -18.1019 to -8.15276 | Yes | **** |
