# Supplementary material for: Acupuncture combined with moxibustion mitigates spinal cord injury-induced motor dysfunction in mice by NLRP3-IL-18 signaling pathway inhibition
Source: J Orthop Surg Res. 2023 Jun 9;18:419. doi: 10.1186/s13018-023-03902-6 (PMC10257262; doi:10.1186/s13018-023-03902-6)
Supplement: Supplementary file 3 — Additional file 3. Gene knockout strategy. [file 13018_2023_3902_MOESM3_ESM.pdf]

A

Product Information

Name	C57BL/6N-Nlrp3^{em1cyagen}
Serial Number	CKOCMP-216799-Nlrp3-B6N-VA
Gene	Nlrp3
NCBI ID	216799
Strain	C57BL/6N
Type	conditional knockout

B

loxp Forward: 5’-TTGTGGAGGATGGGAAGTCTAAAG-3’

loxp Reverse: 5’-CTCAGATAGACACCATCGTCTCAG-3’

Cre Forward: 5’-GAACGCACTGATTTCGACCA-3’

Cre Reverse: 5’-GCTAACCAGCGTTTTCGTTC-3’

C

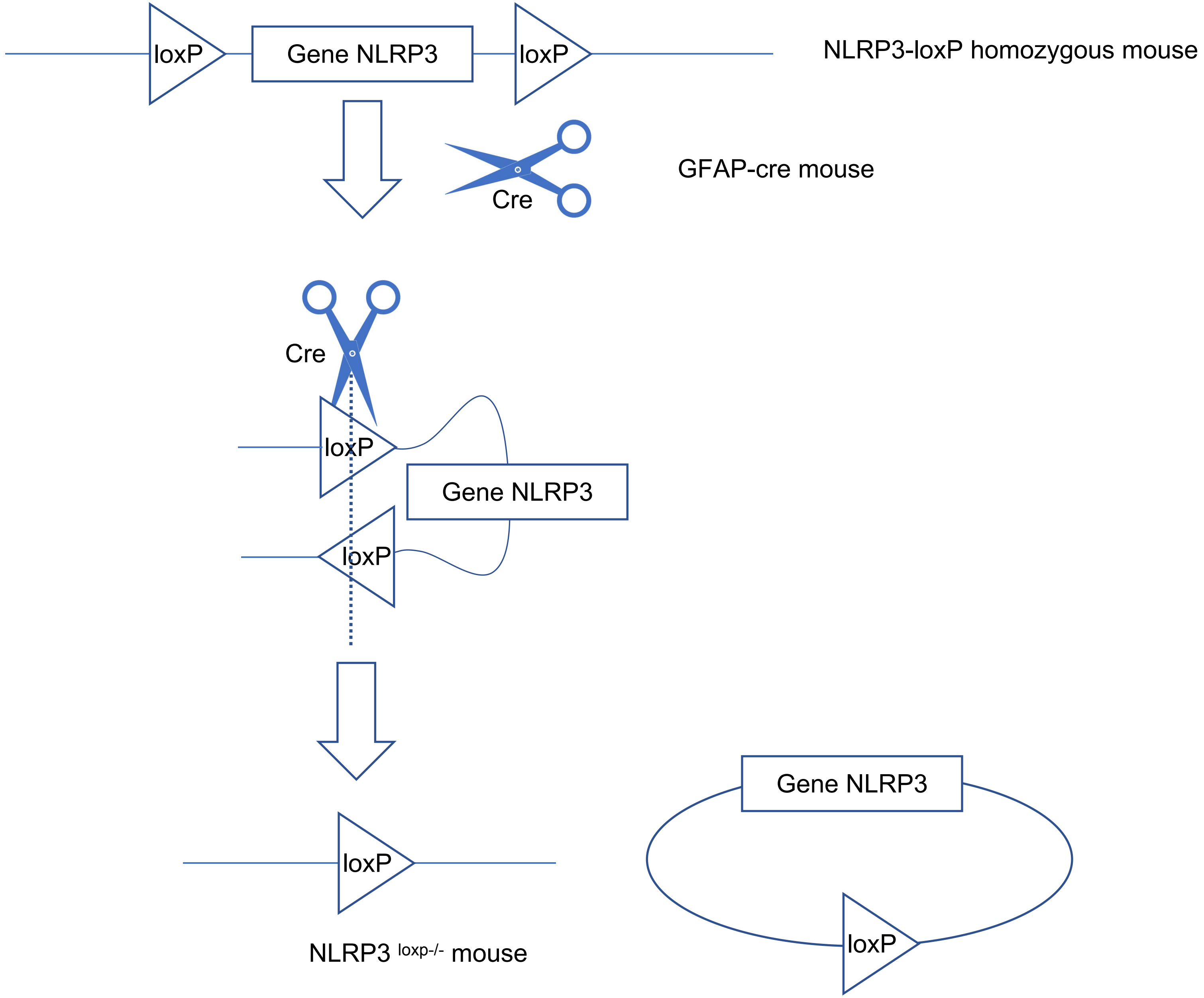

loxP
Gene NLRP3
loxP
NLRP3-loxP homozygous mouse
Cre
GFAP-cre mouse
Cre
loxP
Gene NLRP3
loxP
loxP
Gene NLRP3
loxP
NLRP3 loxp-/- mouse

D

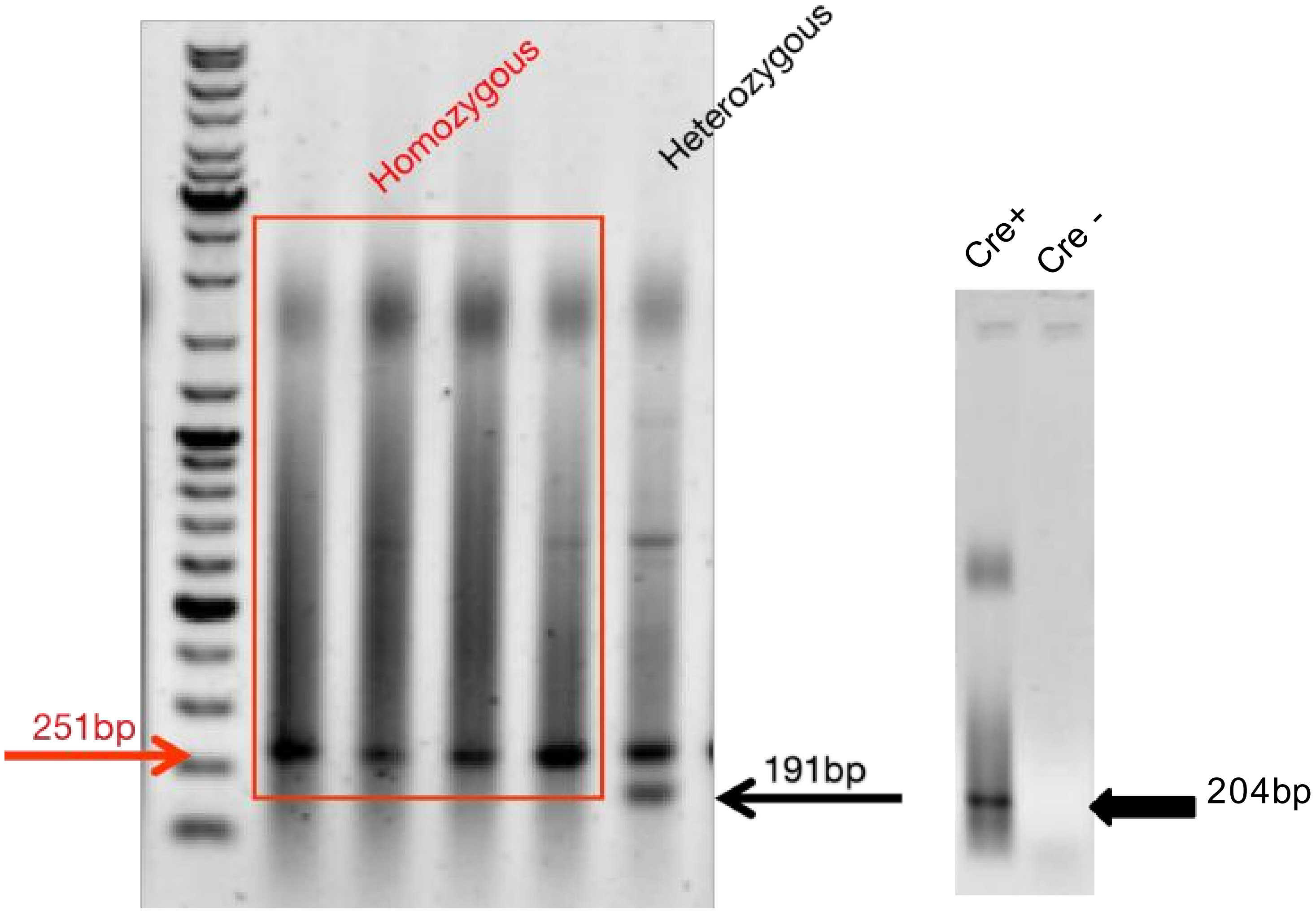

A. NLRP3 Conditional Knockout Mouse Line Product Information B. Primers related to conditional knockout mouse gene identification C. Schematic diagram of the process of knocking out NLRP3 in astrocytes D. Graph of gel electrophoresis results of mouse gene identification by PCR. Homozygous with cre are mice that have knocked out NLRP3 in astrocytes.
