# Supplementary material for: Acupuncture combined with moxibustion mitigates spinal cord injury-induced motor dysfunction in mice by NLRP3-IL-18 signaling pathway inhibition
Source: J Orthop Surg Res. 2023 Jun 9;18:419. doi: 10.1186/s13018-023-03902-6 (PMC10257262; doi:10.1186/s13018-023-03902-6)
Supplement: Supplementary file 4 — Additional file 4. Original western blots. [file 13018_2023_3902_MOESM4_ESM.pdf]

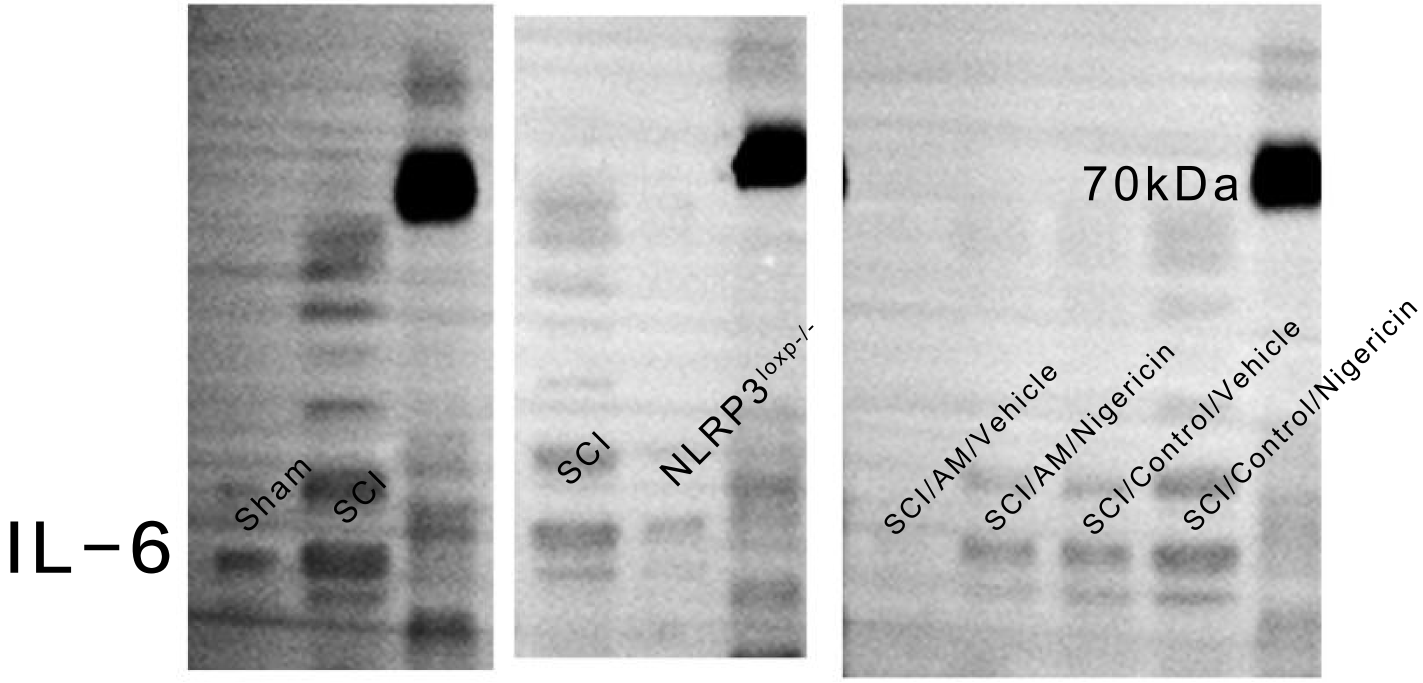

Original western blots for each group of IL-6 proteins

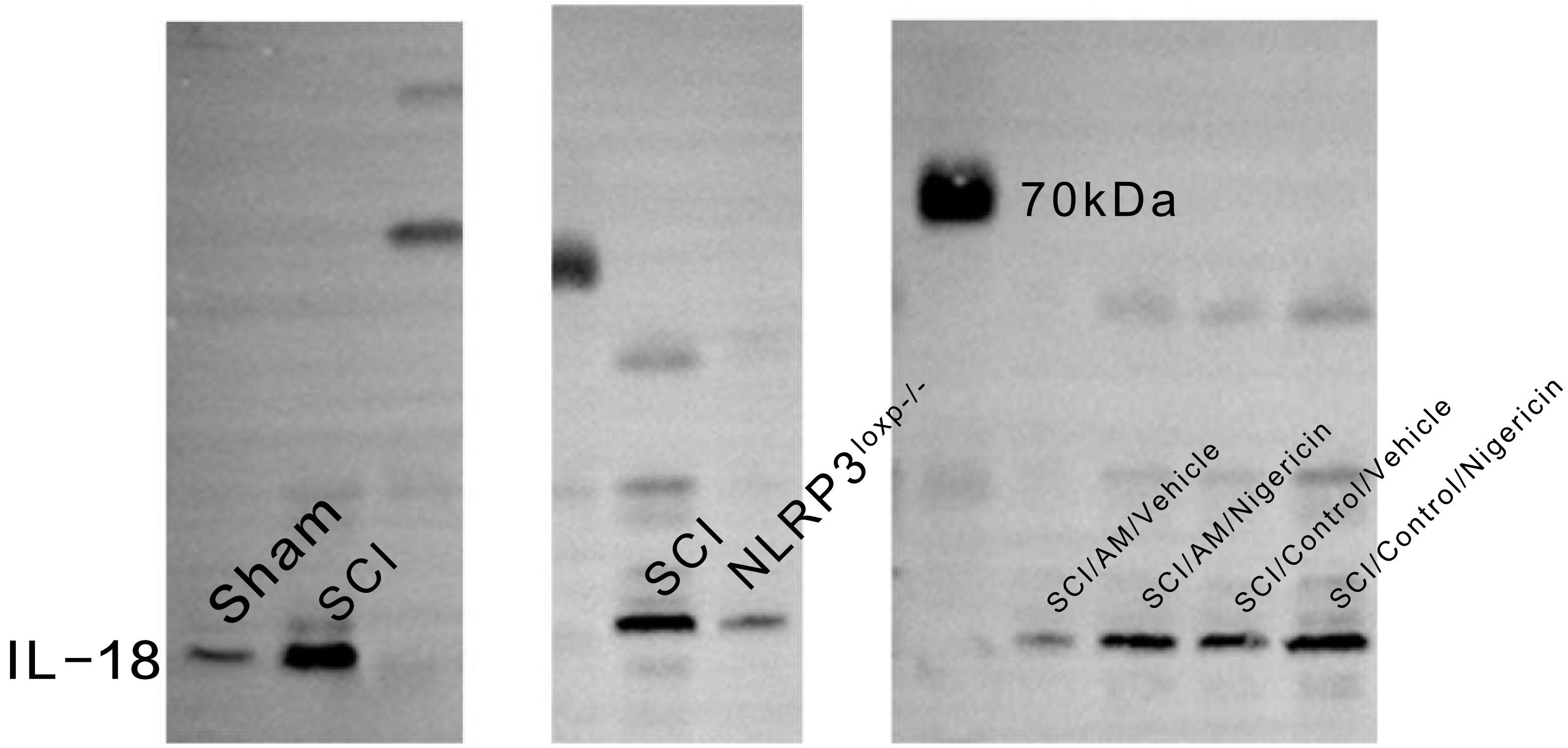

Original western blots for each group of IL-18 proteins

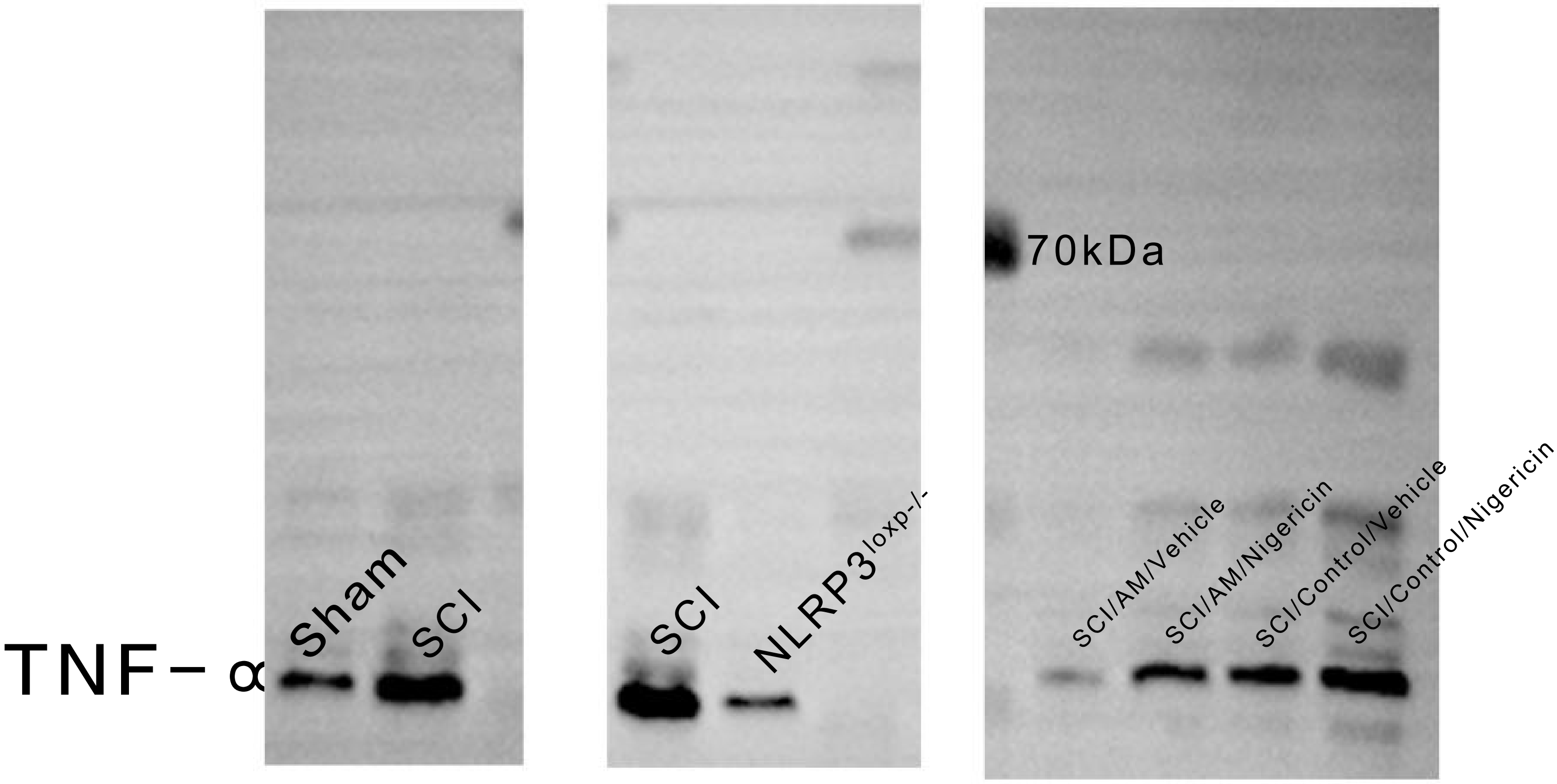

Original western blots for each group of TNF-α proteins

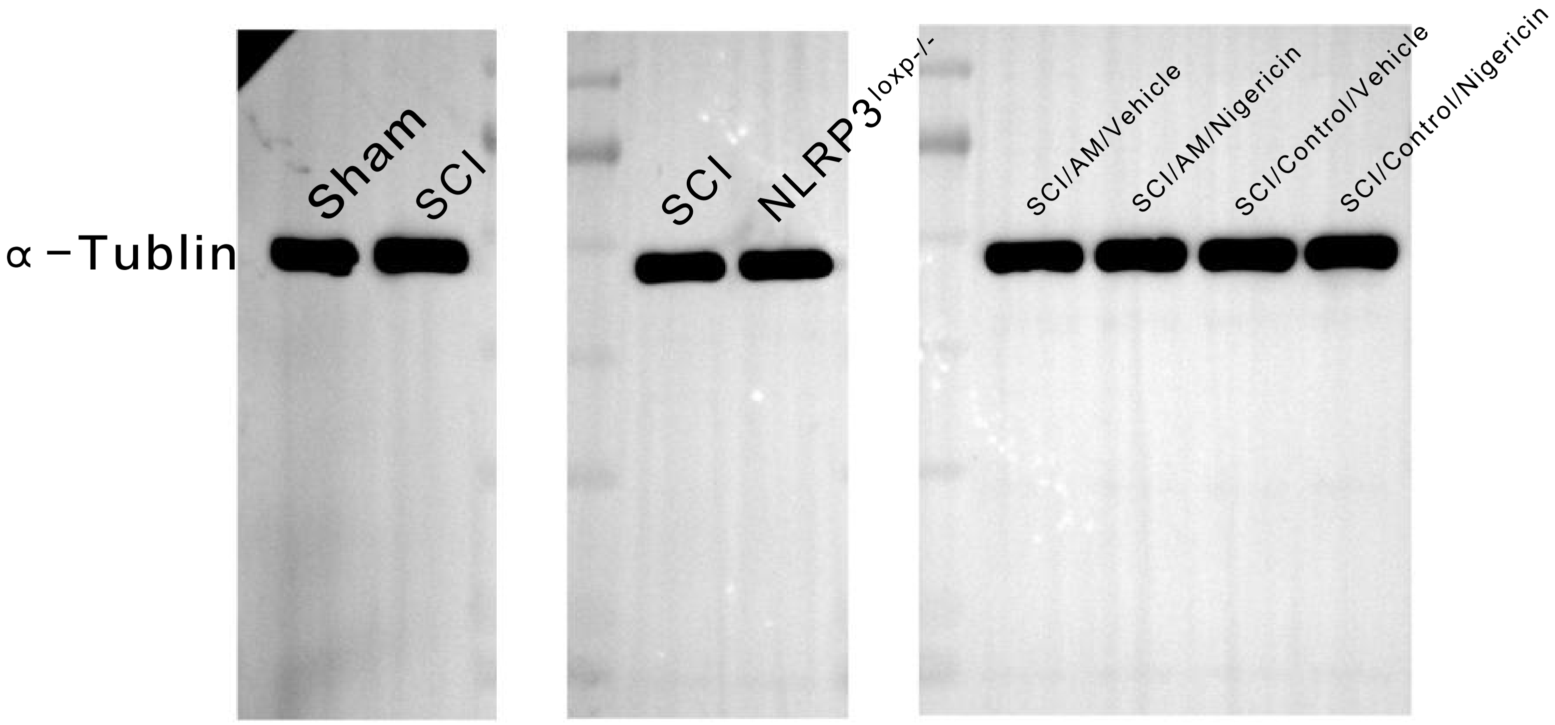

Original western blots for each group of α-ublin proteins
